# Transforming Shiga toxin-producing *Escherichia coli* surveillance through whole genome sequencing in food safety practices

**DOI:** 10.3389/fmicb.2023.1204630

**Published:** 2023-07-13

**Authors:** Stéphanie Nouws, Bavo Verhaegen, Sarah Denayer, Florence Crombé, Denis Piérard, Bert Bogaerts, Kevin Vanneste, Kathleen Marchal, Nancy H. C. Roosens, Sigrid C. J. De Keersmaecker

**Affiliations:** ^1^Transversal Activities in Applied Genomics, Sciensano, Brussels, Belgium; ^2^IDlab, Department of Information Technology, Ghent University—IMEC, Ghent, Belgium; ^3^National Reference Laboratory for Shiga Toxin-Producing Escherichia coli (NRL STEC) and for Foodborne Outbreaks (NRL FBO), Foodborne Pathogens, Sciensano, Brussels, Belgium; ^4^National Reference Centre for Shiga Toxin-Producing Escherichia coli (NRC STEC), Universitair Ziekenhuis Brussel, Vrije Universiteit Brussel, Brussels, Belgium; ^5^Department of Plant Biotechnology and Bioinformatics, Ghent University, Ghent, Belgium

**Keywords:** whole genome sequencing, Shiga toxin-producing *Escherichia coli*, surveillance, food safety, implementation

## Abstract

**Introduction:**

Shiga toxin-producing *Escherichia coli* (STEC) is a gastrointestinal pathogen causing foodborne outbreaks. Whole Genome Sequencing (WGS) in STEC surveillance holds promise in outbreak prevention and confinement, in broadening STEC epidemiology and in contributing to risk assessment and source attribution. However, despite international recommendations, WGS is often restricted to assist outbreak investigation and is not yet fully implemented in food safety surveillance across all European countries, in contrast to for example in the United States.

**Methods:**

In this study, WGS was retrospectively applied to isolates collected within the context of Belgian food safety surveillance and combined with data from clinical isolates to evaluate its benefits. A cross-sector WGS-based collection of 754 strains from 1998 to 2020 was analyzed.

**Results:**

We confirmed that WGS in food safety surveillance allows accurate detection of genomic relationships between human cases and strains isolated from food samples, including those dispersed over time and geographical locations. Identifying these links can reveal new insights into outbreaks and direct epidemiological investigations to facilitate outbreak management. Complete WGS-based isolate characterization enabled expanding epidemiological insights related to circulating serotypes, virulence genes and antimicrobial resistance across different reservoirs. Moreover, associations between virulence genes and severe disease were determined by incorporating human metadata into the data analysis. Gaps in the surveillance system were identified and suggestions for optimization related to sample centralization, harmonizing isolation methods, and expanding sampling strategies were formulated.

**Discussion:**

This study contributes to developing a representative WGS-based collection of circulating STEC strains and by illustrating its benefits, it aims to incite policymakers to support WGS uptake in food safety surveillance.

## 1. Introduction

Shiga toxin-producing *Escherichia coli* (STEC) can cause serious infectious gastroenteritis upon consumption of contaminated food or water, or contact with infected animals or human cases (Barbour et al., 2012). In the past decades, its prevalence has increased for it to become the third or fourth most common cause of bacterial foodborne outbreaks across the European Union [European Food Safety Authority (EFSA), 2019, 2021, 2022; European Food Safety Authority (EFSA), and European Centre for Disease Prevention and Control (ECDC), 2021]. Rapid confinement of STEC outbreaks is of utmost importance to limit their burden.

Whole Genome Sequencing (WGS) has become the tool of choice in the laboratory-based investigations of these outbreaks [EFSA BIOHAZ Panel (EFSA Panel on Biological Hazards) et al., 2019; Nouws et al., 2020a]. It offers an unprecedented resolution to analyze isolate relationships with higher accuracy than ever before. For the same reason, the Centers for Disease Control and Prevention (CDC), the Food and Drug Association (FDA), the World Health Organization (WHO), the European Centre for Disease Prevention and Control (ECDC), and the European Food Safety Authority (EFSA) advice countries worldwide to adopt WGS in routine public health (i.e., clinical isolates) and in food surveillance/monitoring systems as well [World Health Organization (WHO), 2018; European Centre for Disease Control (ECDC), European Food Safety Authority (EFSA) et al., 2019; Food and Drug Administration (FDA), 2022]. As such, WGS is already successfully implemented in a range of countries, like, e.g., the United States (Brown et al., 2019), Canada (Jain et al., 2019), Germany (Revez et al., 2017; García Fierro et al., 2018; News Desk, 2021), the Netherlands (Revez et al., 2017; García Fierro et al., 2018; Friesema et al., 2021), France (Moura et al., 2016; Revez et al., 2017; García Fierro et al., 2018), Denmark (Kvistholm Jensen et al., 2016; Revez et al., 2017; García Fierro et al., 2018) and the United Kingdom (Revez et al., 2017; García Fierro et al., 2018; Rumore et al., 2018). However, in most other countries WGS is currently only used in outbreak investigation and public health surveillance whereas its systematic adoption in routine food safety practices evolves more arduously and is limited to analyzing distinct strains of ongoing outbreaks. As STEC is a One Health problem involving the human-animal-environmental interface (Karmali, 2017), it is, however, important that the same methodology is applied across the different STEC reservoirs, and not kept limited to the human one. Only then, a reliable representation of the circulating background can be obtained to analyze isolate relationships, which is indispensable to enable timely detection of outbreaks. Although National Reference Laboratories (NRLs) of these countries have often built sufficient capacity by now to perform WGS on food isolates and to deliver reliable WGS-based results (García Fierro et al., 2018; Nouws et al., 2020a), the restricted budget granted by their national policymakers is still one of the main restraining factors for the slower WGS uptake in food safety monitoring practices (García Fierro et al., 2018). Most studies demonstrating the benefit of WGS in STEC food surveillance are focussed on countries with larger financial support (Deng et al., 2016; Rumore et al., 2018; Brown et al., 2019; Galarce et al., 2021). Delivering evidence-based arguments on its benefits for countries where WGS is not yet routinely implemented in food surveillance is needed as well to further stimulate national policymakers to support its implementation within these countries.

By virtue of its complete isolate characterization (i.e., serotyping, virulence and antimicrobial resistance (AMR) genotyping), WGS in surveillance is additionally of interest and advised to be used to improve the epidemiological knowledge of STEC. Sufficient WGS data on the diversity and prevalence of different circulating STEC characteristics across different reservoirs is required to define STEC of the highest risk in different countries (i.e., risk assessment) and to attribute these STEC to specific sources (i.e., source attribution), all with the goal of identifying points of attention for effective risk mitigation and control. Moreover, these data can also be used to identify changing trends in circulating STEC, including cross-pathotypes. These are defined as strains harboring the STEC-identifier *stx* gene in combination with pathogenicity genes identifying another *E. coli* pathotype (Koutsoumanis et al., 2020). Because of the plasticity of the *E. coli* genome, there is a continuous risk for emerging strains with new pathogenic features, as was the case for the STEC O104:H4 strain that caused the large outbreak in Germany in 2011 (Frank et al., 2011). Routinely applied methods, often following the International Organization for Standardization (ISO) Technical Specification (TS) 13136: 2012 for STEC detection and identification within food and environmental samples are only focussed on the conventional detection of the major virulence genes (i.e., *stx* and *eae*) and serotypes (O157, O111, O26, O103, and O145; International Organization for Standardization ISO/TS 13136: 2012, 2012) and on *stx-or* serogroup-based STEC isolation via selective media. This further decreases isolation rates or may create a bias (if serogroup-based) and limits the surveillance for other important characteristics. WGS enables the detection of (all) important genetic virulence features when present, rendering it the ideal tool to more comprehensively survey the prevalence of cross-pathotypes and other emerging strains.

Besides following up on trends in circulating strains, the information generated with WGS can support risk assessment when combined with available metadata (Butcher et al., 2016; Chattaway et al., 2016; Mughini-Gras et al., 2018c). Risk assessment is a process to identify potential hazards and analyze what could happen if a hazard occurs (United States Government, 2022). An important aspect in this context is assessing the pathogenicity of STEC strains. Lately, STEC pathogenicity prediction is based on associations determined between the presence of certain virulence genes and *stx* gene subtypes within isolates and the clinical outcome (EFSA Panel on Biological Hazards (BIOHAZ), 2013). The application of a holistic approach, for which WGS constitutes an ideal candidate, is of interest because it allows complete virulence genotyping [EFSA BIOHAZ Panel (EFSA Panel on Biological Hazards) et al., 2019; Koutsoumanis et al., 2020]. With the last ongoing revision of the ISO standards used in routine monitoring, this point of view regarding *stx* gene subtyping is added, including a recommendation to use WGS in the process (International Organization for Standardization ISO/TS 13136: 2012, 2012; Koutsoumanis et al., 2020). However, it was stated that insufficient WGS data on STEC are available to date to perform sound association studies using innovative statistical approaches in combination with detailed phenotypic features of involved cases, leaving important data gaps to be filled on STEC pathogenicity (Koutsoumanis et al., 2020).

In addition to STEC pathogenicity, a combination of other aspects, such as stress response characteristics and antimicrobial resistance (AMR), affects the risk of STEC infection. Any stress slowing down the growth rate of *E. coli* induces the RpoS-controlled general stress response (Battesti et al., 2011). The wild-type *rpoS* gene was hypothesized to be important for STEC survival within the acidic intestines of its principal host, i.e., cattle (Price et al., 2000). On the contrary, *rpoS* mutants were hypothesized to increase the scavenging properties of STEC to gather scarce nutrients and survive in competition with commensal *E. coli* in the human intestinal tract (van Hoek et al., 2013; Franz et al., 2015). However, further research is required to deliver a deeper insight into the role of RpoS in the adaptation of STEC to diverse environments and reservoirs. Investigating the prevalence of *rpoS* mutant STEC strains in different reservoirs with traditional methods would require additional Polymerase Chain Reactions (PCR) for *rpoS* detection followed by Sanger sequencing of the amplicons, which are time-consuming and costly, and are not part of current routine STEC surveillance methods. Thanks to its single nucleotide resolution, WGS in STEC surveillance would enable easily investigating the prevalence of *rpoS* mutants across STEC isolates hypothesis without requiring extra wet-lab experiments.

AMR is an increasing global public health issue, related to the excessive administering of human and veterinary antimicrobial drugs. Although the use of antimicrobials in STEC infection treatments is contraindicative (Agger et al., 2015; Freedman et al., 2016), monitoring AMR in STEC isolates like in any commensal *E. coli* remains important as, from a One Health perspective, these strains contribute to the resistance gene reservoir that is easily exchanged between different bacterial species (Kennedy et al., 2017). In contrast to conventional testing that is targeted to specific antibiotics, WGS enables full AMR phenotype prediction. Moreover, conventional AMR testing is only performed routinely for strains isolated from human cases or in case of outbreaks, whereas WGS in surveillance would enable AMR genotyping for isolates from different reservoirs without the need for extra analyses. Multiple studies have shown good phenotype prediction from the determined genotype for STEC bacteria (Boysen et al., 2014; Lindsey et al., 2016; Vieira et al., 2016).

STEC surveillance also focusses on identifying the different sources that are responsible for causing human disease (i.e., source attribution). Attributing human cases to putative sources of infection is crucial to identify the necessary interventions in the food production chain to limit disease burden (Pires et al., 2009). The so-called frequency-matching source attribution models are commonly used in source attribution studies for multiple foodborne pathogens (Mughini-Gras et al., 2018c) and are based on matching certain isolate characteristics (such as, e.g., serotypes) to different reservoirs (Mughini-Gras et al., 2018b). Since the routinely used conventional methods only target a limited number of specific characteristics of STEC, source attribution studies are often also only focussed on these characteristics. Because WGS enables complete isolate characterization, it allows performing more extensive and complete source attribution studies.

To unlock the full potential of WGS in STEC surveillance, it is however indispensable that mature surveillance systems are in place. Indeed, the reliability of epidemiological studies is dependent on the representativeness of the applied WGS-based dataset. These monitoring systems are defined by extensive samplings, ideally targeting all different STEC reservoirs according to a One Health approach. Microbiological safety of products across the farm-to-fork food chain (i.e., farm production site, slaughterhouse, packing plant, manufacture, and retail) is guarded by conventional analysis of official control (i.e., regulated by the national authorities) and self-checking (i.e., regulated by the food business operators themselves) samples for the detection and isolation of foodborne pathogens such as STEC. Instead of using conventional methods for characterization, sequencing these strains by a laboratory with WGS capacity would enable investigating the complete genotype of isolates (if isolation was successful). A highly mature food safety monitoring system would, in theory, pick up all threats circulating in the farm-to-food chain, enabling WGS to detect any genomic link between clusters of human isolates and related food surveillance isolates. The efficiency of applying WGS in such surveillance systems has been demonstrated previously (Allard et al., 2016; Butcher et al., 2016). However, multiple studies and the EFSA reported that representative datasets are rarely obtained due to biased sample availability [Mughini-Gras et al., 2018a; Thépault et al., 2018; EFSA BIOHAZ Panel (EFSA Panel on Biological Hazards) et al., 2019]. The application of WGS in STEC food surveillance would allow indicating and evaluating the extensiveness of applied surveillance and sampling strategies. Moreover, it would also allow identifying potential gaps and drafting suggestions that can aid in further optimizing these strategies. The EFSA stated that studies defining sampling strategies that ensure statistical power and sufficient representativeness are crucial to bringing WGS in food surveillance to the next level [EFSA BIOHAZ Panel (EFSA Panel on Biological Hazards) et al., 2019].

In this study, a collection of sequenced STEC isolates was created based on the surveillance of STEC across the different reservoirs in Belgium, combined with publicly available data from other studies. This collection was used: (I) To determine the added value of WGS in STEC food safety surveillance for outbreak investigation by the ability to include temporally and/or spatially dispersed isolates; and (II) To evaluate the circulating serotypes, virulence factors, antimicrobial resistance factors, and *rpoS* mutants in STEC strains across different reservoirs in Belgium, which can be important information for future more elaborate source attribution and risk assessment studies. Moreover, suggestions were formulated that can assist in further optimizing sampling strategies and policies, based on the indicated profoundness of the current surveillance system. With this collection, an effort was made to construct a dataset with strains circulating in Belgium that can be used in outbreak prevention and investigation, or in future epidemiological studies. Together with evidence-based input delivered on the benefits of routine WGS-based STEC surveillance, we hope to directly address and stimulate policymakers to support WGS implementation in food safety monitoring, thereby contributing to the recommendations of EFSA and ECDC concerning the application of WGS for foodborne pathogens and the setup of a WGS-based dataset representative for the circulating background that can ultimately be added to the joint EFSA-ECDC database [European Centre for Disease Control (ECDC), European Food Safety Authority (EFSA) et al., 2019].

## 2. Materials and methods

### 2.1. Collection of STEC isolates

A collection of isolates was set up based on strains circulating in Belgium, mainly picked up by the national STEC surveillance system. For isolates to be included in the collection, some predefined selection criteria that are specified in Supplementary material had to be fulfilled. In total, the final collection analyzed in this study contained 754 WGS data sets. The corresponding strains were isolated between 1998 and 2020 (including eight human strains isolated in the beginning of 2021) and originated from various food samples (n: 194), human cases (n: 323), and animal carcasses or feces (n: 237; Supplementary Figure S1). All food and animal strains in this collection originated from samples taken in the scope of national STEC surveillance (i.e., from official control and self-checking samples) or outbreak investigation and were isolated by the NRL of STEC (NRL STEC). All human strains in this collection were sampled from human cases in the scope of public health surveillance and isolated by or centralized at the National Reference Centre of STEC (NRC STEC). For 405 isolates, WGS data were already produced and publicly available on NCBI SRA; i.e., raw reads produced in the scope of different Belgian studies (Nouws et al., 2020a,b; Bogaerts et al., 2021) or on ENA (i.e., produced by the NRC STEC), leaving an additional 349 isolates that were processed and sequenced in-house in the scope of this study. Metadata on the patients’ gender, age at the time of infection, and symptoms (asymptomatic, diarrhea, bloody diarrhea, Hemolytic Uremic Syndrome (HUS), or others) are available for 263 of the 323 human isolates in Supplementary Table S1. This table moreover contains an overview of the different isolates within the collection and their characteristics (sample reservoir, sector (if applicable) and year of isolation). Supplementary Table S2 shows an overview of the number of strains within this collection per isolation year and the reservoir that they were isolated from. For the remainder of this study, isolates originating from animal carcasses or animal feces were defined as coming from the animal reservoir/origin (see Supplementary material), strains isolated from food samples across the complete food chain were defined as coming from the food reservoir/origin, and isolates from human cases were defined under the human reservoir/origin.

### 2.2. Sample preparation and sequencing

The isolates processed and sequenced in this study were preserved in a glycerol-brain heart infusion broth stock (40.0%) at –80°C until the start of the experiments. A loopful of each pure culture stock was grown overnight (16 h at 37°C) on nutrient agar plates. PCR was performed on the majority of the isolates to detect *stx* and *E. coli* housekeeping *uidA* genes (see Supplementary Table S3) on a heat-lysed extract prepared of a single colony. Leftovers of the same colony that was used for the heat lysis was inoculated in 10 mL of BHI and grown overnight with shaking at 37°C and 200 rpm, if STEC presence was confirmed (i.e., *stx* and *uidA* positive). These cultures were used for GenElute Bacterial Genomic DNA (gDNA) extraction (Sigma-Aldrich, Missouri, United States), according to the manufacturer’s instructions, but with DNA elution in 1 M TrisHCl (pH 8.0) or 10 mM TrisHCl (pH 8.5). Each DNA extract was preserved at −20°C until Nextera XT DNA library preparation (Illumina, San Diego, CA, United States) according to the manufacturer’s instructions. The libraries were sequenced on a MiSeq instrument with the MiSeq V3 chemistry (Illumina, San Diego, CA, United States) for the production of 2 × 250 bp paired-end reads.

### 2.3. WGS data analysis

All WGS data were analyzed on an in-house Galaxy instance with the ‘push-button’ STEC pipeline that is also available at the public Galaxy instance of Sciensano (https://galaxy.sciensano.be, registration required; Bogaerts et al., 2021). Shortly, raw reads underwent quality trimming using Trimmomatic 0.38 (Bolger et al., 2014), and genome assembly using SPAdes 3.13.0 (Bankevich et al., 2012), followed by downstream analyses as described below.

#### 2.3.1. Evaluation of dataset quality

Assembly statistics were calculated on the filtered assembly with QUAST 4.4 (Gurevich et al., 2013). The processed reads were mapped against the assembly using Bowtie2 2.4.2 (Langmead and Salzberg, 2012) with the “—end-to-end,” “—sensitive,” and “—phred33” options enabled, and used to estimate the coverage with SAMtools depth 1.9 (Li, 2011). Several quality metrics were then computed, as previously described and defined (Bogaerts et al., 2021). Kraken2 2.0.7 (Wood et al., 2019) was used to check for contaminant reads (i.e., other than *E. coli*) against a database set up in-house, containing all NCBI RefSeq Genome entries (lastly updated on 18th February 2019) for the taxonomic groups archaea, bacteria, fungi, human, protozoa and viruses. Potential raw reads classified as contaminant bacterial species (up to the genus level) were manually removed if detected. More information on the quality control check per isolate can be found in Supplementary Table S4.

#### 2.3.2. Characterization of STEC isolates

Read-mapping based detection of AMR, virulence and serotype determining genes was performed using SRST2 0.2.0 (Inouye et al., 2014) on the trimmed reads with the ResFinder (Zankari et al., 2012; Kleinheinz et al., 2014), VirulenceFinder (Joensen et al., 2014; Kleinheinz et al., 2014) and SerotypeFinder (Joensen et al., 2015) databases. For the analyses with the three databases, hits with <60.0% query coverage and >10.0% divergence (i.e., default settings) were omitted. Database sequences were retrieved from their corresponding sources on 14th November 2021 and clustered on 80.0% sequence identity using CD-HIT 4.6.8 (Li and Godzik, 2006) before use. The VirulenceFinder database that was applied for virulence gene detection enables the detection of different gene alleles, but does not annotate them with their corresponding variant name in the database. Therefore, based on the detected allele, the variant name was manually determined using BLAST with the sequence of the detected virulence gene allele against the standard nucleotide collection (nt) database. When the *stx1* variant of *Shigella dysenteriae* type 13,818 T was called with VirulenceFinder, the workflow described by Bogaerts et al. (2021) was applied to confirm the species of the isolates to be *E. coli*. For recently discovered serogroups whose corresponding *wzx* and *wzy* gene alleles were not yet present within the SerotypeFinder database, reference sequences were obtained from literature (Lang et al., 2019; Supplementary Table S5).

#### 2.3.3. Analyzing phylogenetic relationships between the isolates

Relationships between isolates were investigated through *in silico* core genome Multilocus Sequence Typing (cgMLST) by aligning assembled contigs against the EnteroBase (Zhou et al., 2020) cgMLST scheme (updated on 21st November 2021) using BLAST+ 2.6.0 (Altschul et al., 1990, 1997). All allele calls were accepted in the matrix and no further filtering was done. GrapeTree 1.5.0 (Zhou et al., 2018) was used to construct a minimum spanning tree based on the allele call matrix, with the “method” option set to “MSTreeV2.” The tree was visualized with iTOL (Letunic and Bork, 2019).

#### 2.3.4. Detection of single nucleotide polymorphisms in the *rpoS* gene

To detect Single Nucleotide Polymorphisms (SNPs) in the stress response *rpoS* gene, trimmed reads of bovine animal isolates and of human isolates were mapped against the *rpoS* reference (NC_000913.3 geneID: 947210) with Bowtie2 2.4.2 (Langmead and Salzberg, 2012), using the presets “—sensitive” and “—local.” SAMtools 1.9 (Li et al., 2009; Danecek et al., 2021) and BCFtools 1.9 (Danecek et al., 2021) were used to call variants in the *rpoS* gene. First, pileups were created using the SAMtools mpileup command, with the “—count-orphans” option enabled and the minimum base quality option set to zero. Afterwards, variants were called with BCFtools call command, with the “—ploidy” parameter set to one and other options left at default values. Both SNP and indel variants were called. No other variant filtering was done. The novel consensus sequences were then extracted using the BCFtools consensus command with default settings. CLC Sequence Viewer 8.0 was used to do a local alignment for the extracted *rpoS* consensus sequences of all human isolates, and for those of all animal isolates of bovine origin. The average percentages of obtained numbers of variants were compared between both reservoirs. The potential effect of these SNPs and indels on the corresponding amino acid sequence was also investigated by translating the consensus DNA sequences using CLC Sequence Viewer 8.0 and by searching for the mutations in scientific literature.

### 2.4. Epidemiological study of STEC in Belgium and indication of profoundness of national STEC surveillance system

To deliver insight on the different STEC strains circulating in Belgium, the diversity of virulence and AMR characteristics and serotypes was described across the complete collection, i.e., determined on the collection of 754 WGS data sets based on strains isolated between 1998 and 2020 (including those isolated in the beginning of 2021), irrespective of the different reservoirs. Any prevalence study was performed on a part of the collection being representative of the circulating STEC strains in Belgium, i.e., containing the majority of isolates obtained from the surveillance system for a specific reservoir (e.g., when comparing between reservoirs, the isolates from 2018 to 2020 were included). More detailed information concerning the performed epidemiological studies and used sub-collections can be found in Supplementary material and Supplementary Table S6.

The Chi-Squared test, a non-parametric statistical test, was used similarly as before (Bugarel et al., 2010b; Buvens et al., 2012; Franz et al., 2015; De Rauw et al., 2018b; Bai et al., 2021) to investigate whether an association exists between the presence of certain virulence genes or gene alleles and the incidence of severe disease [defined as the cases that developed HUS (n: 48)]. Only the human isolates for which metadata concerning their clinical state during disease were available were used to investigate associations of different STEC characteristics with severe disease (i.e., 263 of the in total 323 human isolates, see Supplementary Table S1). The Chi-Squared test was only applied when expected values were bigger than five and when sample sizes were bigger than 30. The null hypothesis was rejected when obtained value of ps were <0.05, i.e., a significant result is obtained indicating an association. The statistical analyses were performed using the R programming language 4.2.1 (R Core Team, 2019).

## 3. Results

### 3.1. WGS in STEC food surveillance to benefit the detection of genomically close background isolates, including spatially or timely dispersed ones, and to resolve outbreaks

**Figure 1:**
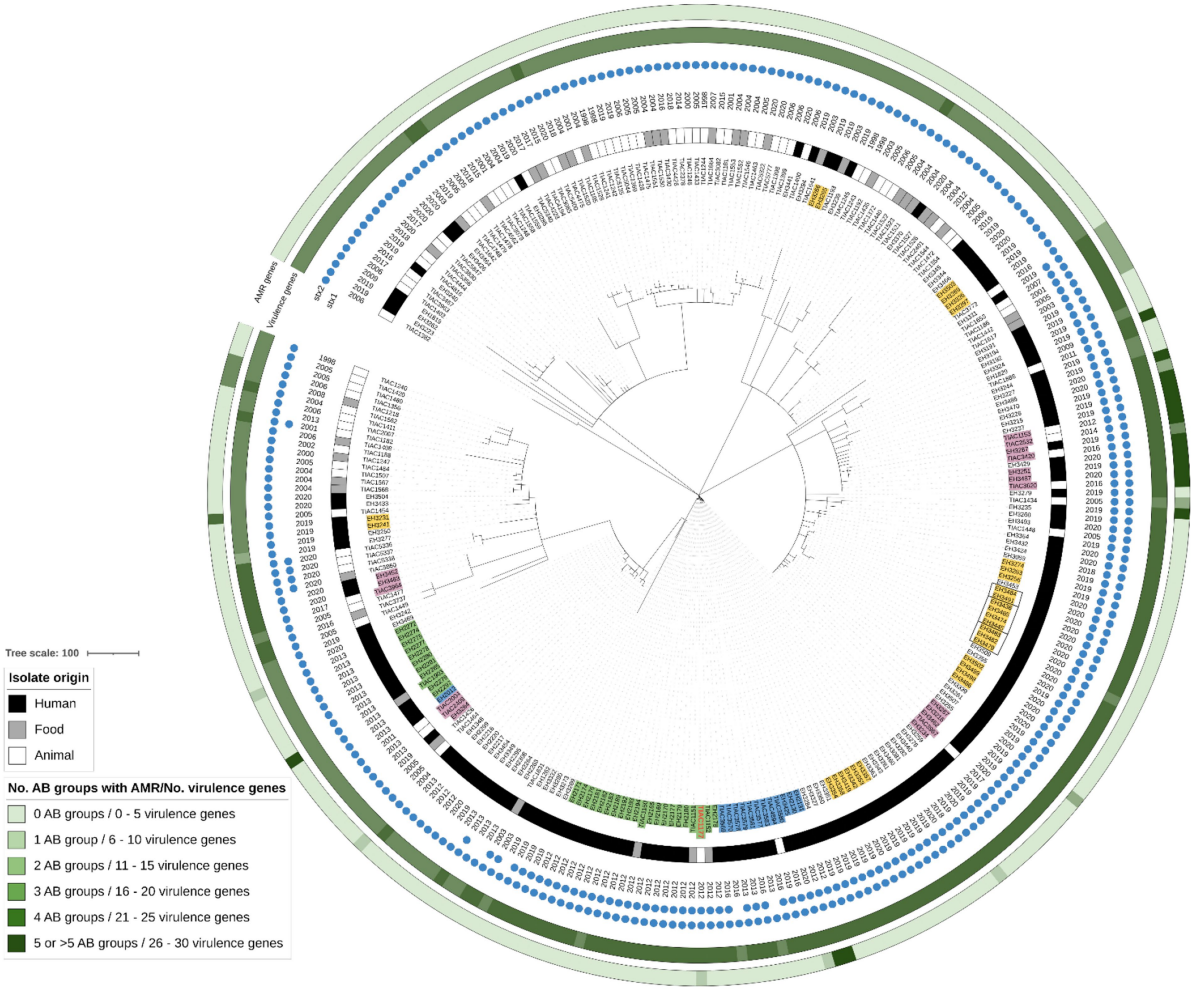
cgMLST tree of all O157 human, food and animal isolates within the complete STEC collection. A minimum spanning tree was created based on the cgMLST allele matrix of all isolates using the MSTreeV2 method with GrapeTree. The tree was then visualized with iTOL and the O157 isolates were pruned in the tree visualized here. The origin of the isolates (i.e., corresponding legend), year, presence of *stx1* and *stx2* and the number of virulence genes and antibiotic (AB) groups for which AMR was predicted (i.e., corresponding legend) are annotated on the tree. The scale bar represents the number of core genome allele differences. Isolate names belonging to the Limburg outbreak (2012) and the Flanders outbreak (2013) are marked in light green. The TIAC1177 isolate detected in this study to cluster with the Limburg outbreak is shown in bold red font. Isolate names marked in light blue are closely linking back to the outbreak clusters. Isolates marked in light purple indicate clusters (n: 5) of human isolates for which a link with the food/animal reservoir was detected. Isolates marked in yellow indicate clusters (n: 9) of human isolates for which no link with the animal/food reservoir could be detected.

The STEC collection contained all isolates sampled by the official controls. Isolates with serotype O157:H7 accounted for a large fraction of the collection (n: 277 isolates out of 754). A cgMLST tree containing all O157:H7 human, food and animal isolates across the collection was visualized in Figure 1. In total, seven clades of human isolates clustering together with an isolate of animal or food origin picked up by the food safety monitoring were identified across the O157:H7 lineage. Two of these clades concerned two previously described outbreaks in Belgium: (I) The Limburg 2012 outbreak caused by a STEC *stx1*+, *stx2*+, *eae* + O157:H7 strain belonging to the Hierarchical cgMLST cluster HC5 number 15809 [i.e., HC5_15,809, as determined by Enterobase (Zhou et al., 2020)] in filet Americain that could be confirmed with conventional methods (Braeye et al., 2014; Nouws et al., 2020a); and (II) The Flanders 2013 outbreak caused by a STEC *stx2*+, *eae* + O157:H7 strain in Americain filet for which no epidemiological evidence was found and conventional methods were not discriminatory enough to define the outbreak cluster (Nouws et al., 2020a). Both outbreaks were previously analyzed in the scope of a study to evaluate the benefit of WGS in outbreak investigation (Nouws et al., 2020a). For the Limburg 2012 outbreak, the application of the large dataset of background isolates in this study enabled the identification of one extra bovine carcass isolate (TIAC1177; Figure 1: isolate name with bold red text and marked in light green), dating from 1 month after the start of the outbreak, to cluster together with the conventionally determined outbreak cluster consisting of 17 human isolates and three food isolates (Figure 1: isolate names marked in light green). All isolates within this outbreak cluster differed by a maximum of two cgMLST alleles. TIAC1177 had an identical virulence genotype as the outbreak strain, but differed in its AMR profile with conferred resistance against β-lactams. Moreover, 13 human and one different animal carcass isolates were identified to be closely clustering with the outbreak based on core genome similarities (Figure 1: isolate names marked in light blue). Besides two that had no *stx1* gene and three that had a different AMR gene profile compared to the outbreak strain, all others had identical virulence and AMR gene profiles. These strains had been isolated a few months before and multiple years after the outbreak (Supplementary Table S7A, i.e., 2013, 2016, and 2019 and 2020) and differed by five, six, eight, nine, 11, 12 or 22 cgMLST alleles from the outbreak strain. Except for EH2130 and EH2131, which were already previously detected to be genomically similar to the outbreak strain (Nouws et al., 2020a), the number of cgMLST allele differences with the outbreak strain was directly proportional to the time dispersed between the isolation of the background isolate and the outbreak.

Also the five other clades of human and animal or food isolates contained both temporally dispersed isolates (with a maximum of 6 years difference) as well as strains consecutively isolated within a short time period (i.e., the same year) that were genomically similar. None of the isolates within a single cluster contained more than seven cgMLST allele differences (Supplementary Table S7B). These genomic isolates’ relationships were not detected previously with conventional methods in the scope of routine STEC surveillance. Moreover, within each cluster, all isolates had an identical virulence and AMR gene profile, except for one cluster consisting of two isolates in which predicted resistance against β-lactam and sulphonamides was lost in the more recent isolate. Multiple similar clusters could moreover be detected in other serotype lineages (as an example, the clusters discovered in the other serogroups prevalently observed across the human collection are shown in Supplementary Figure S2: isolate names marked in light purple).

In addition to these clusters containing both human and food or animal isolates, nine other clusters consisting of only human isolates were identified within the O157:H7 lineage (Figure 1: isolate names marked in light yellow). None of these clusters contained an isolate picked up by the food surveillance system. Also in other serotype lineages, similar clusters were identified (as an example, the clusters discovered in the other serogroups prevalently observed across the human collection are shown in Supplementary Figure S2: isolate names marked in light yellow).

### 3.2. WGS to elaborate on STEC epidemiology to assist source attribution and risk assessment

#### 3.2.1. Epidemiology of STEC serotypes

Conventional routine serotyping that targets O26, O103, O111, O121 (only for human isolates), O145 and O157 would have left 40.2% of the isolates in the collection with unknown serotype, whereas WGS enabled complete serotyping (i.e., O-and H-typing) for 94.0% of the isolates (n: 709 out of 754 isolates). Of the 45 isolates for which serotyping did not succeed, 25 had high sequence similarity between O-type determining genes making them impossible to distinguish using WGS (i.e., O118/151, O90/127, O128ab/ac, O125ab/ac, O123/186, O9(a), and O153/178; Joensen et al., 2015; DebRoy et al., 2016), 14 isolates had an untypeable H-type, five isolates were O-untypeable and one isolate was determined to contain two O-types on the same contig [as previously described by Bogaerts et al., 2021]. In total, a diversity of 78 serotypes were detected across the complete collection (Supplementary Table S8).

**Figure 2:**
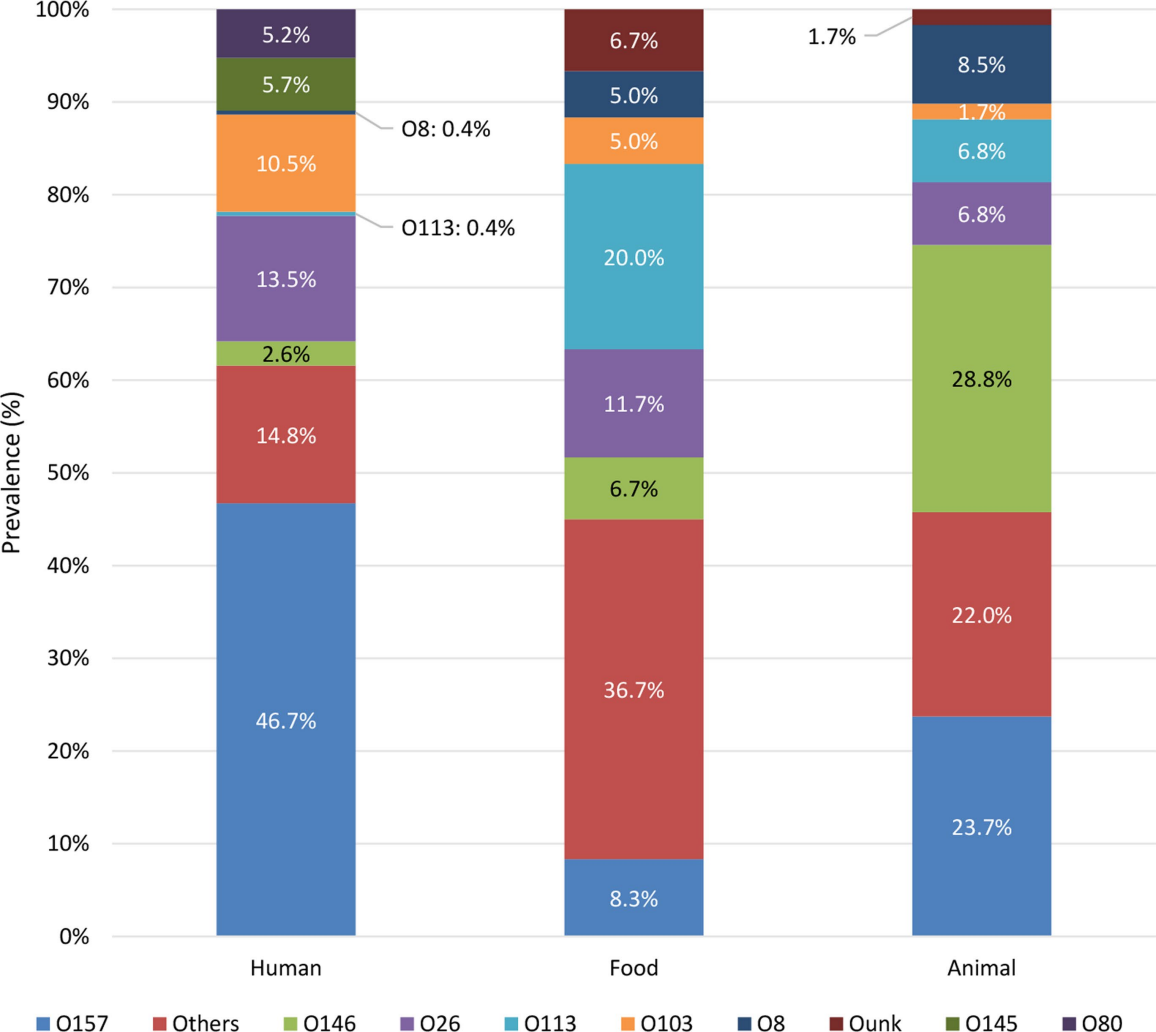
Prevalence of STEC serogroups in each reservoir. Only the prevalence of the top five serogroups in each reservoir is shown, the prevalence of other serogroups is grouped in “Others.” Supplementary Figure S3 visualizes the prevalence of each detected serogroup per reservoir.

The prevalence of serogroups was compared between the reservoirs (summary in Figure 2 and complete overview in Supplementary Figure S3). The serogroups most frequently observed across the human isolates in the collection were O157, O26, O103, O145, and O80. Across the food reservoir, the main serogroups circulating were O113, O26, O157, and O146. Moreover, isolates with serogroup left unknown (i.e., Ounk) also accounted for 6.7% of the isolates across the food collection. O26 was observed to be the main serogroup circulating in the food reservoir in the initial years (i.e., 2014 to 2015, Supplementary Figure S4A). However, O26 was replaced by the emerging O113 serogroup in almost all succeeding years until 2019. In 2020, O26 as well as O157 were observed to be the main serogroups within the food reservoir. Animal isolates within the collection were mainly of serogroups O146, O157, O8, O113, and O26. Over the years, different serogroups with similar prevalences were observed to circulate in the first 3 years for which representative data for the animal reservoir were available, with a slightly increased frequency of O5, O118/151 and O113 in 2014, 2015, and 2016, respectively (Supplementary Figure S4B). However, starting from 2015, the number of detected O157 and O146 isolates started to increase, reaching the highest prevalence across the animal reservoir from 2017 onwards.

**Figure 3:**
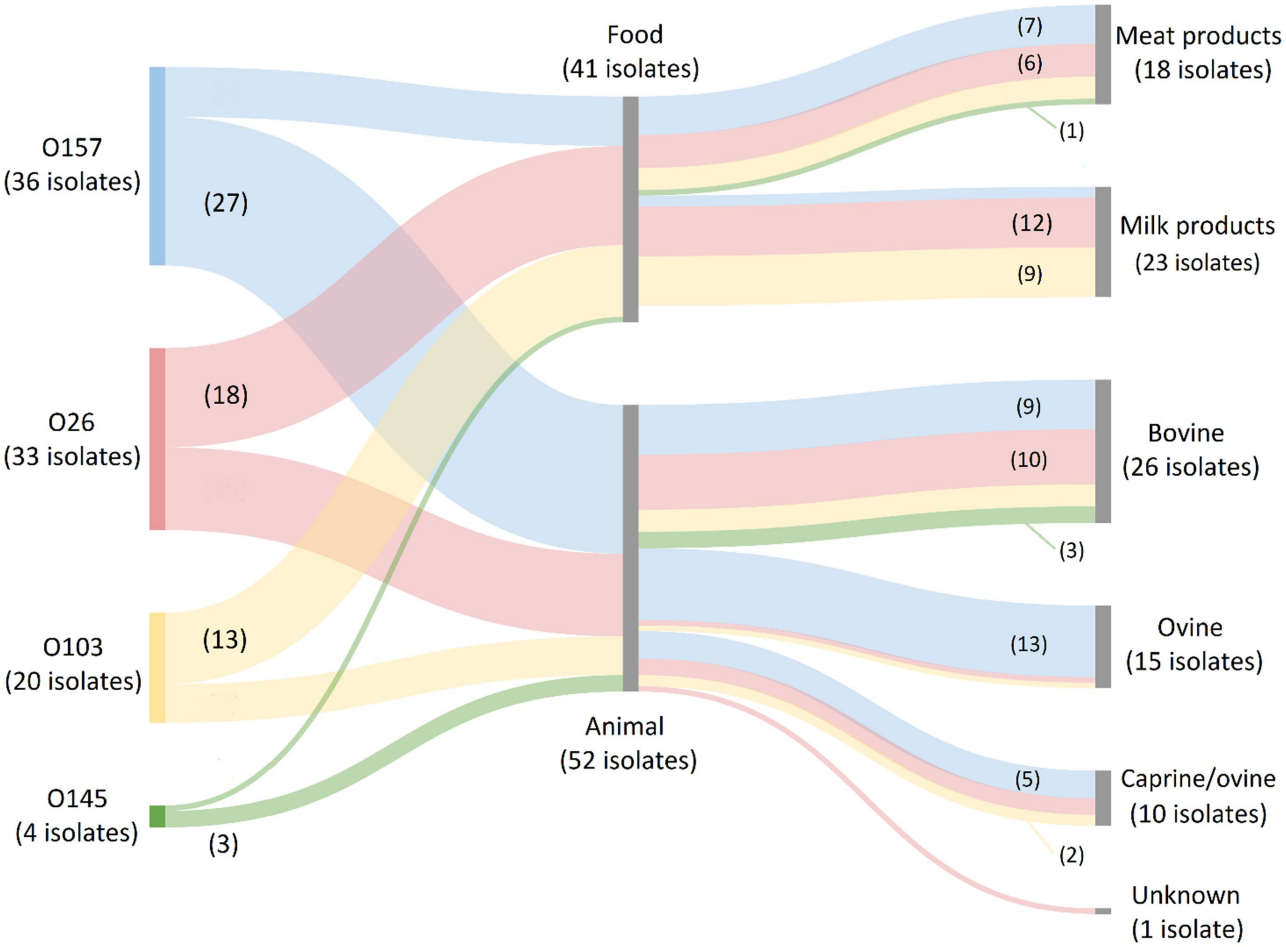
Prevalence of O157, O26, O103, O145, and O80 (the most frequent serogroups in human disease) in the food and animal reservoirs. The Sankey diagram was made with SankeyMATIC (https://sankeymatic.com/build/) and attributes were added manually with Microsoft Paint. The colored flows are serogroup-specific and visualize the number of isolates with that specific serogroup in each food and animal reservoir. The number of isolates with a specific serogroup in each sample reservoir is shown or can be deduced. Note that O80 isolates are not present in the diagram because they were not detected across the food and animal reservoirs. Additionally, no STEC of the top 5 serogroups were isolated from vegetables.

For the serogroups most frequently observed across human isolates in our collection (i.e., O157, O26, O103, O145, and O80), it was investigated in which food or animal reservoirs they could mainly be detected. Sources within the food reservoir were subdivided into: (I) Meat and derivatives; (II) Milk and dairy products; or (III) Vegetables. Sources within the animal reservoir were subdivided into: (I) Bovine; (II) Ovine; (III) Ovine/caprine; or (IV) Unknown origin. The results are shown in Figure 3. O157 isolates were mainly represented in the animal reservoir (in 16.3% of the animal isolates), but also across the food reservoir (in 5.8% of the food isolates). Across the animal reservoir, 48.2% of the O157 isolates originated from ovine carcasses (and/or feces), whereas 77.8% of the O157 strains within the food reservoir were isolated from bovine meats or derivatives. O26 and O103 isolates were represented across the food (in 11.7% and 8.4% of the food isolates, respectively) and animal reservoir (in 9.0% and 4.2% of the animal isolates, respectively). Of the O26 and O103 isolates picked up from food samples, 66.7% and 69.2%, respectively, were of milk or dairy origin (mainly of unknown species) and 33.3% and 30.8% respectively, were meats of bovine origin. Across the animal reservoir, 66.7% and 57.1% of the O26 and O103 were detected in bovine isolates. Serogroups O145 and O80 were, respectively, only scarcely and completely not represented within the animal and food collections. Overall, the food reservoir mainly containing the most prevalent serogroups in Belgium was milk or dairy products of unknown (47.8%), bovine (30.4%), or caprine (21.7%) origin. The main animal reservoir containing these serogroups was bovine (46.3%), ovine (37.3%) or ovine/caprine (16.2%) origin.

**Figure 4:**
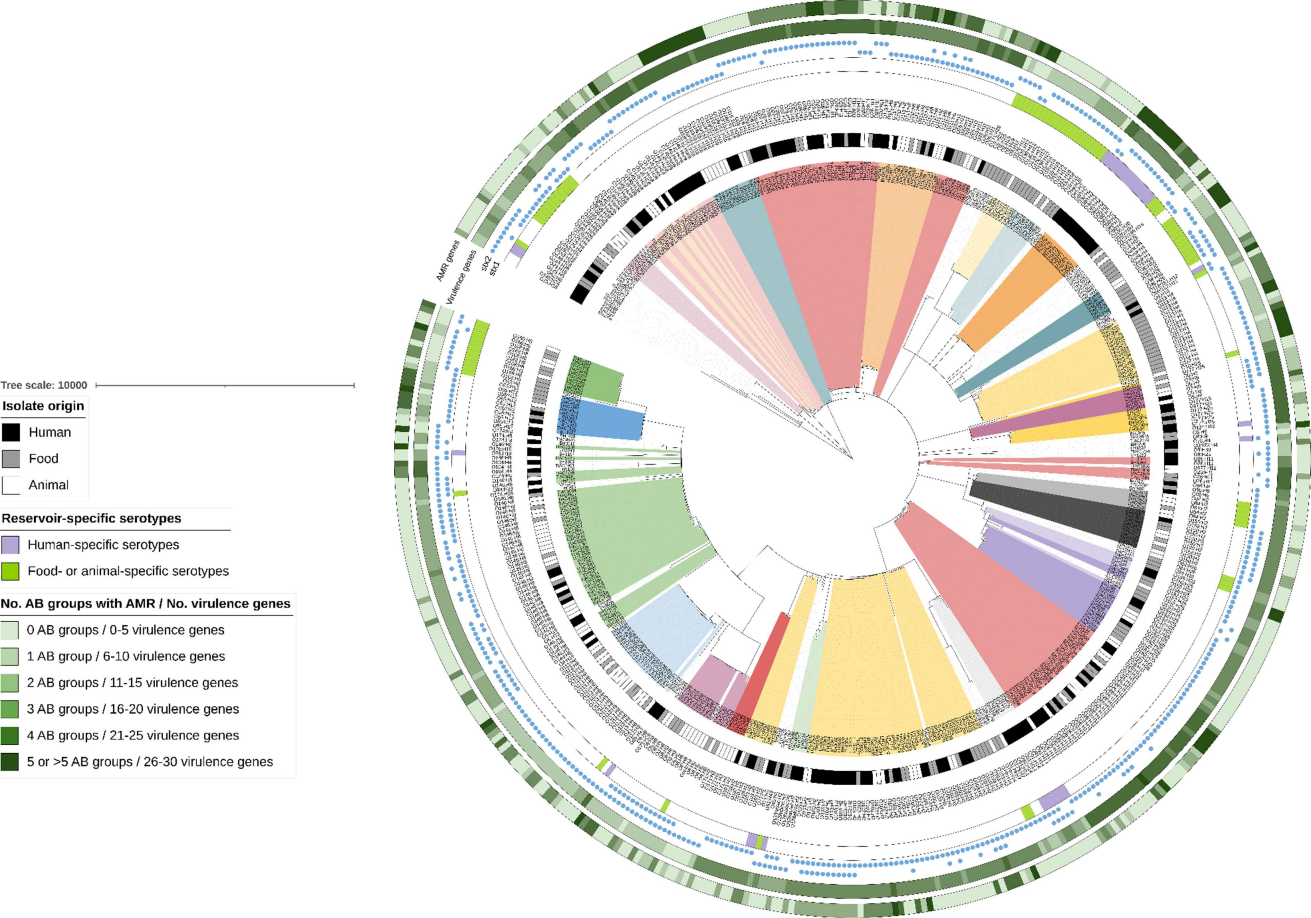
cgMLST tree of all non-O157 isolates within the STEC collection. A minimum spanning tree was made based on the cgMLST allele matrix of all isolates in the collection using the MSTreeV2 method with GrapeTree. The tree was then visualized with iTOL (Letunic and Bork, 2019) and all non-O157 isolates were pruned in the tree visualized here. The O157:H7 lineage was not included to maintain clarity of the phylogenetic tree (to see isolate relationships between the O157:H7 isolates, see Figure 1). The scale bar represents the number of core genome allele differences. Different annotations were added to the tree. From inner to outer circle: (I) Isolate origin (i.e., corresponding legend); (II) Serotypes (colored ranges were moreover added to all isolates with serotypes that had a prevalence higher than 0.5%); (III) Reservoir-specific serotypes indicated with colored strips (i.e., corresponding legend); (IV) presence of *stx1* and *stx2*, as indicated with the colored blue circles. The last two annotations indicate the number of virulence genes and the number of antibiotic (AB) groups for which AMR was predicted (i.e., corresponding legend). Darker colors indicate an increasing number of virulence genes and increasing number of AB groups for which AMR was predicted.

As visible in the phylogenetic tree in Figure 4, isolate clustering mainly occurred according to serotype. Some reservoir-specific serotypes were observed. Indeed, 30.4% of the detected serotypes were only represented in the animal and food reservoir (Figure 4: bright green in color strip concerning reservoir-specific serotypes). Moreover, 17.9% of the serotypes were only detected across the human reservoir (Figure 4: bright purple in color strip concerning reservoir-specific serotypes), a few of which had a prevalence of more than 1.0% across our collection of human isolates (e.g., O80:H2—5.2% and O91:H4—1.7%).

#### 3.2.2. Epidemiology of STEC virulence genotypes

Whereas the conventional methods for routine characterization of food safety surveillance isolates only target the presence of *stx* and *eae* genes according to ISO/TS 13136:2012, WGS enables detection of the complete virulence genotype of isolates. Of the 139 virulence genes present in the VirulenceFinder database that was used for the analysis of the STEC collection, a diversity of 66 virulence genes were detected across all isolates (Supplementary Table S9). Aside from the *stx* gene, the *eae* gene was frequently detected across all STEC isolates. Of all STEC isolates containing the *eae* gene, most of them were observed to be more prevalent in isolates containing the *stx1a*, the *stx2a*, and/or the *stx2c* gene and were of the *eae-γ1* subtype (Supplementary Figure S5). Besides the *stx* and *eae* genes, fiveteen of the 66 detected virulence genes (*astA*, *ehxA*, *espA*, *espB*, *espF*, *espJ*, *espP*, *gad*, *iha*, *iss*, *katP*, *nleA*, *nleB*, *nleC*, *tccP*, and *tir*) were predominantly present across the collection (i.e., in more than 50.0% of isolates). However, differences per reservoir were observed. Two extra virulence genes (*etpD* and *toxB*) were found to be present in more than 50.0% of the human isolates. On the contrary, only 13 (*eae*, *ehxA*, *espA*, *espF*, *espJ*, *espP*, *gad*, *iha*, *iss*, *lpfA*, *nleA*, *nleB*, and *tir*) and five (*ehxA*, *gad*, *iha*, *iss*, and *lpfA*) predominantly present virulence genes were observed across the animal and food isolates in the collection, respectively.

**Figure 5:**
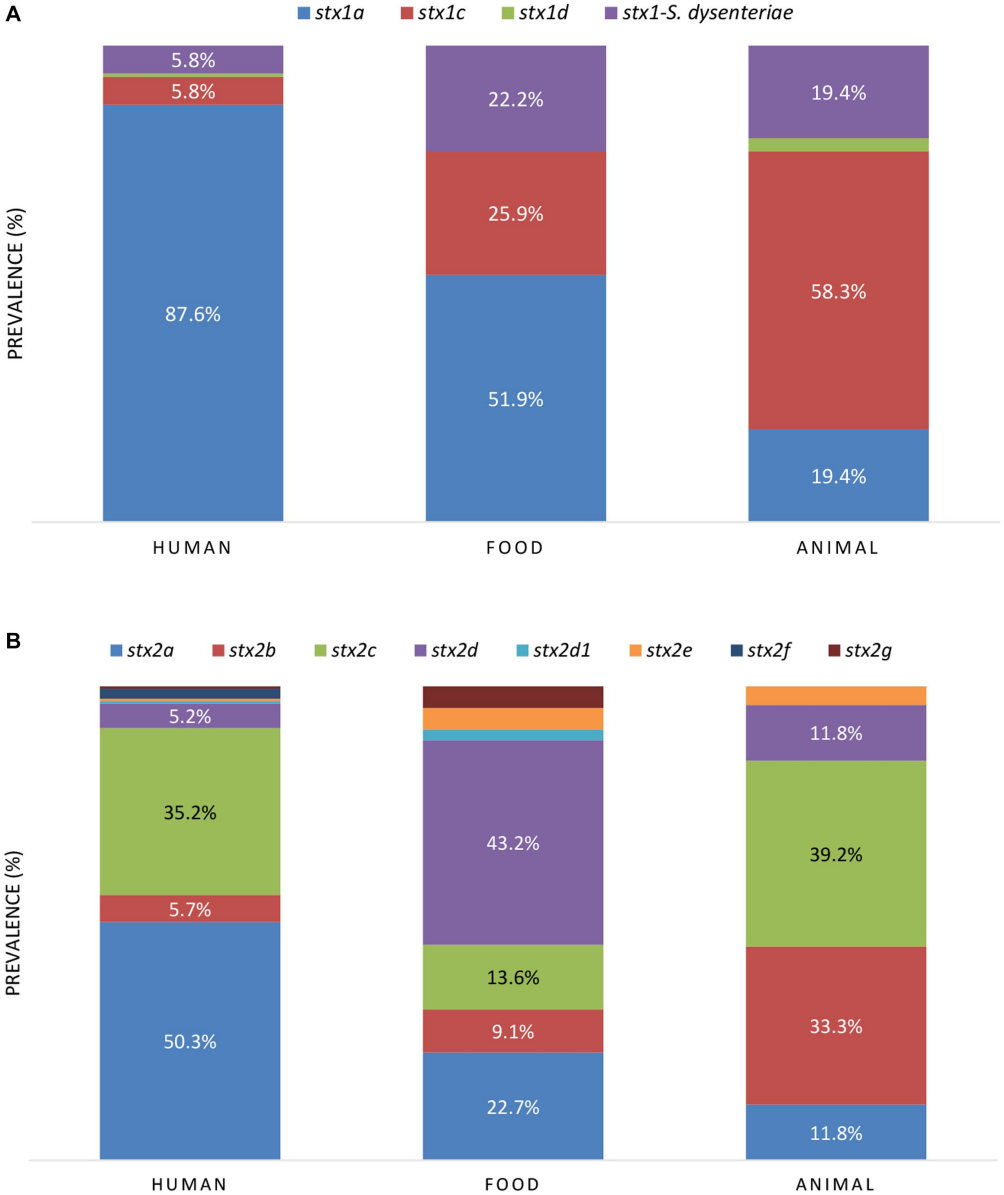
The prevalence of *stx1* **(A)** and *stx2* **(B)** subtypes between the reservoirs. When the prevalence of *stx* variants was higher than 5.0%, the percentages are indicated in the figure.

Across the reservoirs, the sole presence of *stx2* was the most prevalent (42.5%), followed by the joint presence of *stx1* and *stx2* (39.9%), and the sole presence of *stx1* (17.5%). When looking at the *stx* prevalence per reservoir (Table 1), the joint presence of *stx1* and *stx2* was the most prevalent across the human and animal reservoirs, followed by the sole presence of *stx2* and *stx1*, respectively. Across food isolates, the sole presence of *stx2* was more prevalent than the sole presence *stx1*, followed by the joint presence of *stx1* and *stx2*. Of all isolates carrying *stx1*, variant a was the most frequent *stx1* variant across the human and food reservoirs, and variant *stx1c* across the animal reservoir (Figure 5A). Variant *stx2a* was the most frequent in human isolates, *stx2d* in food isolates, and *stx2c* was dominantly found in animal isolates (Figure 5B).

**Table 1:** Prevalence of *stx* genes across the different reservoirs.

Reservoir	Total no. isolates	*stx1*		*stx2*		*stx1* and *stx2*	
		No. isolates	Prevalence (%)	No. isolates	Prevalence (%)	No. isolates	Prevalence (%)
Human	229	37	16.2%	92	40.2%	100	43.7%
Animal	59	8	13.6%	23	39.0%	28	47.5%
Food	60	16	26.7%	33	55.0%	11	18.3%

Because of the high diversity of virulence genes that was detected, the prevalence of STEC cross-pathotypes was also evaluated, based on the detection of virulence identifier genes of other pathotypes (Koutsoumanis et al., 2020; Supplementary Table S10). EPEC-STEC (71.6%) and ExPEC-STEC (17.8%) were the most prevalent, while ETEC-STEC and EAEC-STEC were limitedly prevalent across the reservoirs in the collection (i.e., with 1.4% and 0.6%, respectively, Supplementary Figure S6). No EIEC-STEC and DAEC-STEC were detected. Because of the high prevalence of *eae* (i.e., virulence identifier gene of EPEC) in human isolates, the frequency of EPEC-STEC was the highest within the human reservoir (i.e., 89.1%). In addition, EAEC-STEC were only detected across human isolates, albeit in very low frequencies (i.e., 0.9% or in two out of the 229 isolates). ETEC-STEC were only detected within the food reservoir (i.e., in 8.3% or in five of the 60 isolates). ExPEC-STEC could be detected in all reservoirs, but with the highest prevalence in the animal reservoir (in 39.0% or 23 of the 59 isolates) compared to the human and food reservoir (i.e., in 13.5% and 13.3% of the human and food isolates, respectively).

#### 3.2.3. Association of STEC with severe disease

The prevalence of the different serotypes in severe disease was investigated. In total, 12 different serotypes were observed across the severe human cases (Supplementary Figure S7). Of the detected serotypes, O145:Hunk (i.e., Hunk standing for H-unknown), O104:H4, O113:H4, O150:H2, and O45:H2 were the only one’s more prevalent in severe cases than in less severe cases, albeit that the low number of isolates of these serotypes across the human sub-collection affects the representativeness of their outcome. Moreover, when comparing the prevalence of serotypes in severe disease, all detected serotypes were found to be more prevalent in severe disease than the O157:H7 serotype (11.9%).

It was further investigated if potential associations between the severity of disease and the presence of virulence genes existed based on our collection. The prevalence of different virulence genes in human cases with severe disease, i.e., HUS (n: 48), vs. less severe disease (n: 215) is shown in Supplementary Table S11.

**Figure 6:**
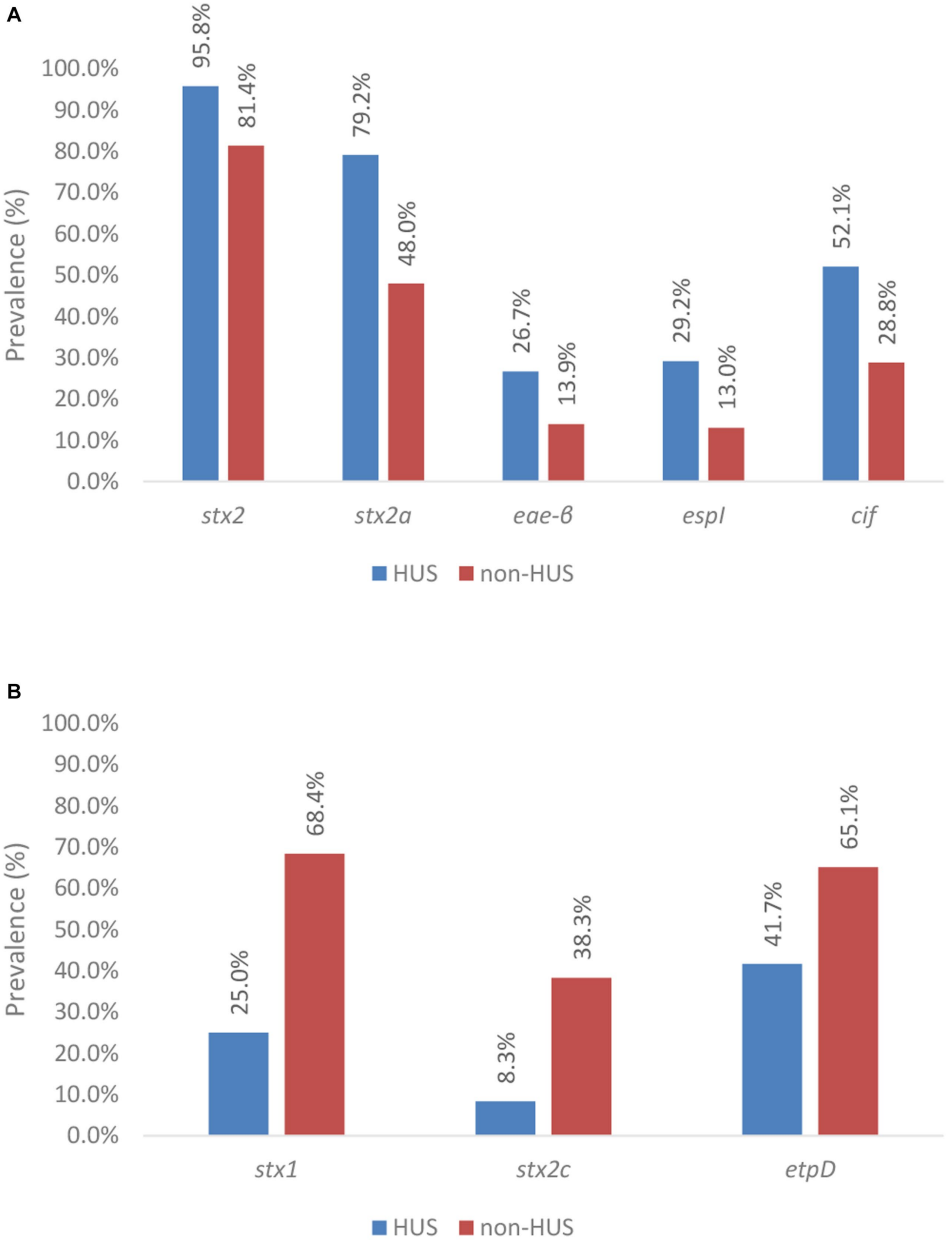
Prevalence of virulence genes across severe (i.e., HUS) and less severe (i.e., non-HUS) disease. Virulence genes and gene variants significantly associated with severe disease are shown in **(A)**, whereas those with a significant association with less severe disease are shown in **(B)**. Statistical tests were based on the Pearson’s Chi-Squared test for which more information about the obtained value of ps can be found in Table 2.

Of the virulence genes detected across the sub-collection (n: 47), 76.6% or 36 genes were more prevalent in HUS cases than in isolates that caused less severe symptoms, i.e., *stx2, eae, iha, ehxA, efa1, astA, espA, espB, espF, espI, espJ, espP, nleA, nleB, nleC, lpfA, katP, tccP, cif, tir, toxB, aggA, aggB, aggC, aggD, aggR, pic, sepA, aaiC, aap, aar, aatA, capU, cba, cma*, *and iroN* (Supplementary Table S11 with gene names in green). The prevalence of genes (variants) for which a significant association with severe or less severe disease was obtained is shown in Figure 6, whereas accompanying Chi squared and value of ps are indicated in Table 2.

**Table 2:** Chi-squared value and *p*-values of virulence genes/gene variants for which a significant association with severe **(A)** and less severe **(B)** disease was determined.

A	Gene allele	Pearson’s Chi-squared value (χ^2^)	*p*-value	B	Gene allele	Pearson’s Chi-squared value (χ^2^)	*p*-value
*stx2*		6.1	1.4E − 02	*stx1*		30.9	1.0E − 05
*stx2a*		17.6	2.7E − 05	*stx2c*		14.6	1.3E − 04
*eae-ß*		4.3	3.8E − 02	*etpD*		9,1	2.6E − 03
*espI*		7.6	5.8E − 03				
*cif*		9.6	2.0E − 03				

The *stx1* gene was observed to be negatively associated with severe human disease. Indeed, *stx1* was only detected in 25.0% of the isolates that caused HUS (n: 12), compared to in 68.4% of the isolates that caused less severe disease. Across all HUS isolates, only the stx1a variant was detected. However, in most of these cases (n: 10 of the 12), *stx1a* was accompanied by the presence of *stx2*. Only in two human HUS cases, a solely *stx1*-positive isolate was isolated. Besides the *stx1a* variant, others (*stx1c, stx1d*, and the *stx1* variant of *Shigella dysenteriae* type 1 strain 3,818 T) were also detected across the isolates that caused less severe disease.

The presence of *stx2* was significantly associated with severe illness. Indeed, *stx2* was detected in 95.8% of the isolates within the HUS subgroup, compared to 81.4% of the isolates within the non-HUS sub-group. Moreover, differences in the prevalence of *stx2* variants across HUS and non-HUS cases were observed. Virulence gene *stx2a* was significantly more prevalent in isolates causing HUS (i.e., 79.2%) vs. less severe disease (i.e., 48.0%). In contrast, the *stx2c* gene was negatively associated with HUS, i.e., it was significantly more prevalent in non-HUS cases. Insufficient data (i.e., expected values were lower than 5) were available to investigate statistical significance of the prevalence of *stx2* variants *d, e, b, f*, and *g*.

Although virulence gene *eae* was observed to be more frequently detected in isolates that caused HUS (i.e., 93.8% and 87.0% of the isolates in the HUS vs. non-HUS sub-group, respectively), no statistically significant association with severe disease was found. Across the isolates that had caused HUS in their corresponding cases, *eae-τ, eae-ξ, eae-γ1, eae-ζ, eae-ε1*, and *eae-β* variants were detected, whereas *eae-α2, eae-α5, eae-θ2*, and *eae-ρ* variants were additionally detected across the collection of less severe cases. Insufficient data were available to perform sound investigations on the associations of severe disease with all these *eae* variants, except *eae-γ1* and *eae-β*. The *eae-γ1* variant was most frequently observed in HUS cases (i.e., 51.1%). However, the variant had an even higher prevalence in non-HUS cases (i.e., 62.6%). No association with severe illness could therefore be determined. The *eae-β* variant was the second most prevalent *eae* variant detected in HUS cases (i.e., 26.7%). For this variant, a significant association with severe illness was observed. Across the isolates causing HUS, only three *eae*-negative isolates were detected. For two of these three isolates, another adherence gene could be identified (i.e., *aggR* and *lpfA*). Of all other virulence genes with a higher prevalence in severe compared to less severe cases in our collection, a statistically significant higher prevalence in HUS cases was only identified for *espI* and *cif*. Insufficient data (i.e., expected values were lower than 5) were available to investigate statistical significance for the higher prevalence of *iha, ehxA, cba, subA, cma, capU, aggA, aggB, aggC, aggD, aggR, iroN, pic, sepA, aaiC, aap, aar*, and *aatA* in severe disease.

Aside from the 36 virulence genes more prevalent in severe disease, 11 other virulence genes were detected across the sub-collection. The *gad* virulence gene was detected in all isolates, irrespective of the clinical outcome of the corresponding human case. All 10 other virulence genes were more prevalent in non-HUS cases, but only the *etpD* gene was determined to be significantly associated with this clinical outcome.

#### 3.2.4. Epidemiology of AMR genotype in STEC isolates

WGS enabled identifying the complete AMR genotype and predicted phenotype of all isolates, whereas AMR phenotyping is routinely only performed on human isolates for a limited set of antibiotics (i.e., ampicillin, cefotaxime, chloramphenicol, ciprofloxacin, gentamycin, kanamycin, nalidixic acid, streptomycin, sulphonamides, tetracycline, and trimethoprim). Antimicrobial resistance was investigated at the antibiotic (AB) group level for all isolates across the collection. In total, a diversity of 63 different AMR genes was detected across the complete collection conferring resistance against 10 different antibiotic groups (i.e., aminoglycosides, macrolides, β-lactams, sulphonamides, tetracyclines, trimethoprims, phenicols, phosphomycins, colistins, and fluoroquinolones, Supplementary Table S12).

All isolates across the complete collection contained the *mdf(A)* gene. This gene encodes an efflux pump that is intrinsically present in *E. coli* bacteria and was therefore not further considered. Across the reservoirs, 67.0% of the isolates was predicted to be susceptible to all AB groups. When comparing AMR between the different reservoirs, the highest prevalence of predicted AMR resistance was observed in the food reservoir (i.e., 43.3% of the food isolates), whereas predicted AMR resistance was lower in the human and animal reservoirs (i.e., 32.3% and 25.4% of the human and animal isolates, respectively). The overall prevalence of resistant isolates across the collection was observed to be decreasing in the last few years (Supplementary Figure S8). However, differences were observed between the reservoirs (Supplementary Figure S9). The prevalence of AMR in the human reservoir was declined steeply, while that in the food and animal reservoirs was, respectively, stable and slightly decreasing.

**Figure 7:**
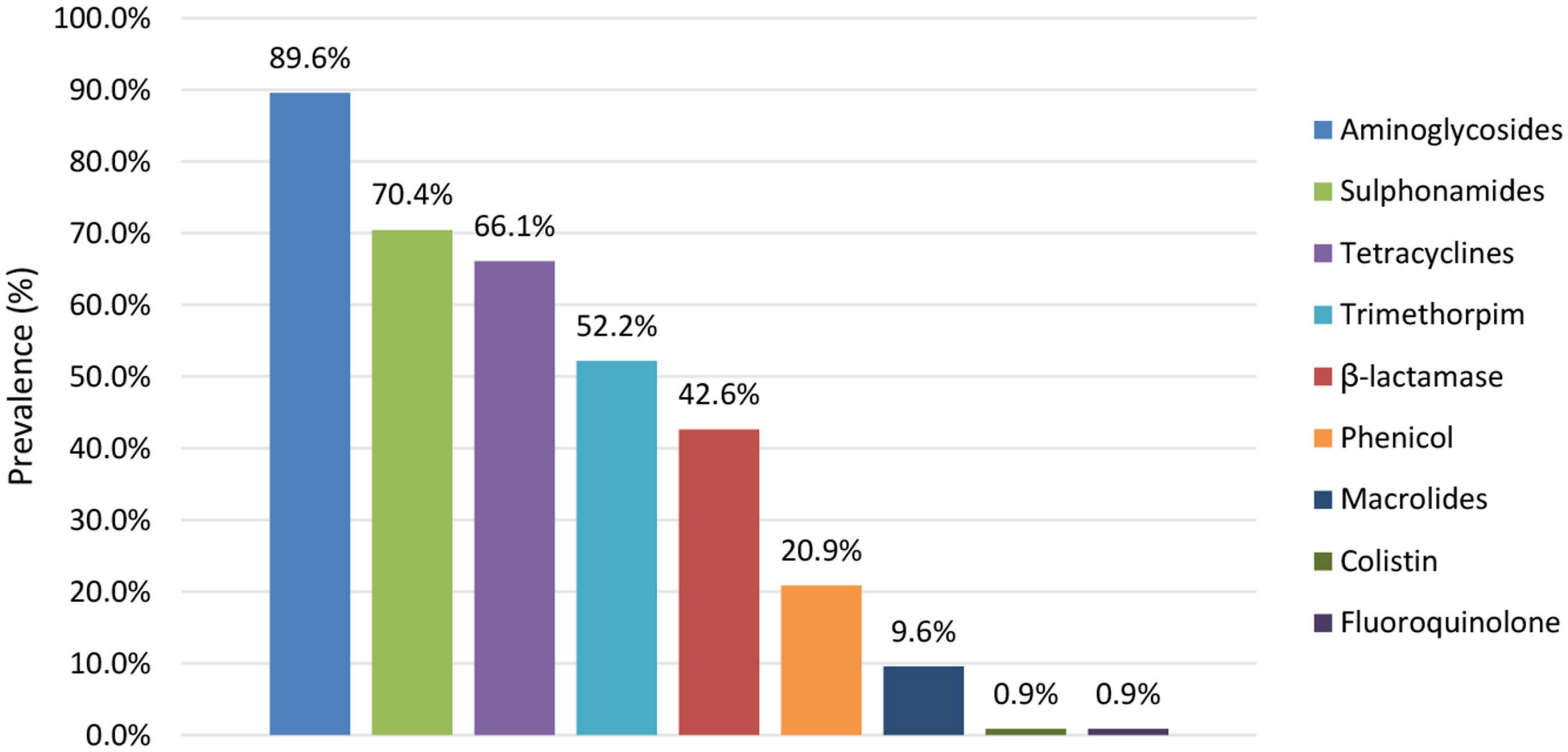
Prevalence of AMR per antibiotic group detected across the reservoirs.

The prevalence of predicted AMR per antibiotic group is shown in Figure 7. Across the isolates with predicted resistance, this was mainly against aminoglycosides (largely conferred by *aph(6)-Id* and *aph(3”)-Ib*), sulphonamides (largely conferred by *sul2*), tetracyclines (largely conferred by *tetA*), trimethoprims (largely conferred by *dfrA1*) and β-lactams (largely conferred by *blaTEM-1B*). These AMR patterns were similar across the different reservoirs. Moreover, all isolates within the collection with predicted resistance against β-lactams were determined to be Extended Spectrum β-Lactamase (ESBL) producing, i.e., containing *blaTEM*, *blaCTX-M*, and/or *blaOXA* genes. Of the isolates with predicted AMR resistance, 88.0% was identified to confer resistance to more than one antibiotic group, including all ESBL-producing isolates. Also when looking at each reservoir separately 93.3%, 84.1%, and 84.7% of the human, food and animal isolates with predicted AMR resistance, respectively, were determined to be multidrug resistant (MDR).

The prevalence of predicted resistance against antibiotic groups was observed to differ between different serotypes (Figure 4; Supplementary Table S13). For 50.0% of the serotypes detected across the STEC collection, prevalence of predicted AMR was higher (such as in, e.g., O26:H11 with 57.9%, O80:H2 with 100.0%, O103:H2 with 55.6%, O113:H4 with 87.5% and O145:Hunk with 30.8% of the isolates for which AMR resistance was observed) than those detected in O157:H7 isolates (i.e., 12.3%). The other serotypes had predicted AMR prevalences lower than those of O157:H7 isolates (e.g., 11.9% of the O146:H8 isolates and none of the O182:H25 isolates had predicted AMR). However, many of these serotypes with lower predicted AMR prevalence were also very scarcely represented across the STEC collection and therefore calculated AMR prevalences should be interpreted with caution.

#### 3.2.5. The single nucleotide resolution of WGS enables to investigate the occurrence of SNPs in interesting genes, e.g., the *rpoS* gene across human vs. bovine isolates

Other studies (van Hoek et al., 2013; Franz et al., 2015) have hypothesized that *rpoS* mutants are important to increase its scavenging properties to look for scarce nutrients in the human intestinal tract, whereas wild-type *rpoS* strains would be of importance in STEC survival in cattle. To investigate whether *rpoS* mutants are indeed more prevalent in human vs. animal isolates from bovine species, the prevalence of each SNP and indel in the *rpoS* gene was identified across the bovine animal and human isolates (Supplementary Table S14). The extracted *rpoS* sequences were also translated to give a first indication of the potential influence of these variants at protein level. Compared to the reference sequence, the *rpoS* C97G SNP was detected in all bovine and human isolates. However, since this SNP is naturally found in non-K12 *E. coli*, it was not further taken into account. In total, more variants could indeed be detected across the human isolates (64.7%, 211 of the 326 isolates) compared to the bovine isolates (58.5%, 83 of the 142 isolates). The majority of these variants (80.8% and 75.0% of the variants identified in the human and bovine isolates, respectively) were synonymous, i.e., without changing the encoded amino acid of the RpoS protein. For only four human isolates, a variant was identified that affected the eventual amino acid sequence, i.e., C266T, G368A, G840T, and A967G. Across the bovine isolates, three isolates were identified to contain a variant in *rpoS* that affects the amino acid sequence, i.e., T296G, G548T, and G554CATGG. The latter mutation concerned an insertion of four nucleotides causing a frame shift. This disrupted triplet reading frame shift results in an early STOP codon and therefore a truncated RpoS protein.

## 4. Discussion

Although WGS is already routinely applied for STEC surveillance in public health surveillance (i.e., for clinical isolates), its implementation in food safety monitoring remains challenging across multiple European countries (Revez et al., 2017; García Fierro et al., 2018), often because of limited financial resources. We performed a retrospective study involving WGS in food safety surveillance for STEC covering the farm-to-food continuum in Belgium and complemented it with WGS data from public health surveillance and other Belgian studies. The complete collection used in this study covered WGS data of 754 strains isolated between 1998 and 2020. Together with the efforts of building a dataset of circulating strains, the goal was to convince and stimulate the national policymakers of countries not yet routinely applying WGS to (financially) support its implementation in STEC food safety surveillance by demonstrating its benefits in the first place in detecting genomically close isolates, even when they are spatially and timely dispersed, and in STEC epidemiology and how this information can eventually be used in future quantitative risk assessment and source attribution studies. Moreover, based on the observations coming from data of the current surveillance system, suggestions on how to further optimize sampling strategies and policies are formulated, as also stated by the EFSA to be important to fully exploit the benefits of WGS.

Thanks to its unprecedented resolution and the possibility to include historical data coming from earlier surveillance efforts, WGS improves linking isolates from sporadic cases and/or contaminated food products, whether or not spatially or timely dispersed, to outbreaks. In this study, besides the isolates previously defined to be part of the Limburg outbreak cluster (Nouws et al., 2020a), one additional isolate was detected to cluster together with the outbreak. This strain was isolated from a bovine carcass by the official control samplings in a slaughterhouse 34 days after the start the outbreak but when the outbreak was still ongoing, although it was not included in the initial outbreak investigation (Braeye et al., 2014). No further metadata on the isolate was available in this study, limiting the further search for epidemiological evidence that is indispensable to confirm and support this genomic link. However, if this genomic link and its accompanying metadata would have been available during the initial investigation, it could have initiated new and directed investigations that potentially might have enabled detecting the cause of the outbreak sooner and identifying how the implicated meat source got contaminated. Additional strains of both human and animal carcass origin, isolated after the outbreak period, were identified to be similar to the outbreak strain at core genome level. Although these genomic similarities should be further investigated at epidemiological level including for instance geographical data, their occurrence might indicate that the outbreak strain was still circulating and potentially causing human disease after the outbreak, as it was likely not removed properly from the food chain. Moreover, similarly as to both outbreaks, different clusters of human cases were discovered with WGS to be genomically related to isolates picked up by food safety surveillance. These links were missed with conventional methods. Between the isolates that are genomically similar and clustered together, an increasing number of cgMLST allele differences was observed between those that are more dispersed over time (i.e., with years in between their isolation date). Although epidemiological investigations are still required to find support for the genomic links in this study, we confirm, as also demonstrated before (Besser et al., 2018; Schürch et al., 2018), that it is better to not use strict thresholds of differences in cgMLST alleles or isolation date for defining isolates as genomically close. Moreover, we agree that these relationships should only be interpreted with the accompanying metadata before defining them as being part of the outbreak. Also in this study, the detected genomic links require epidemiological investigation to accurately define clusters, as otherwise one risks detecting unrelated but genomically close isolates. With the knowledge of these detected genomic links, WGS in food safety surveillance has the potential to guide future investigation in specific directions to retrieve the origin of the outbreak and its accompanying epidemiological evidence, by including timely and/or spatially dispersed isolates in the analysis based on a WGS database in combination with all generated STEC epidemiological data. Although WGS is already applied in public health surveillance, we demonstrated in particular its importance in food safety surveillance as well to improve outbreak investigation.

By providing complete genomic characterization of STEC isolates, WGS in surveillance has moreover the potential to broaden the epidemiological knowledge on circulating STEC strains and strengthen quantitative risk assessment studies. As a proof of concept, the benefits of WGS in risk assessment and source attribution studies were demonstrated in this study, by investigating associations between severe disease outcome and the presence of virulence genes in respective STEC isolates, and by attributing the top five serotypes causing human disease to specific food reservoirs. In the future, once WGS is fully implemented in STEC food safety surveillance and genomic data are routinely generated, it is of interest to perform extensive follow-up risk assessment and source attribution studies by using innovative statistical approaches in combination with detailed phenotypic features of involved cases and specialized modeling approaches that can handle the large amount of genomic data and the discriminatory power associated with it, respectively. WGS allows to detect almost all serotypes [some cannot be distinguished using WGS (Joensen et al., 2015)], whereas conventional methods used by the NRL and NRC STEC that play a major role in national STEC surveillance only allowed to, respectively, detect the European top five (i.e., O26, O103, O111, O145, and O157; Nouws et al., 2020a) or top six (i.e., O26, O103, O111, O121, O145, and O157; Fratamico et al., 2017; Nouws et al., 2020a) serotypes that are known to be most commonly linked to human infection. The serogroups most frequently detected by WGS across the collection of Belgian human isolates between 2018 and 2020 (i.e., O157, O26, O103, O145, and O80; Crombé and Piérard, 2021) differed only slightly from the European top five within the same period [i.e., O157, O26, O103, O146, and O91; European Centre for Disease Prevention and Control (ECDC), n.d.].^1^ Aside from O157, O26, and O103, both O145 and O146 were determined to be important serogroups in our collection of human isolates as well as in other countries worldwide (Brooks et al., 2005; Tseng et al., 2016; Friesema et al., 2020; Koutsoumanis et al., 2020; Loconsole et al., 2020; Ramstad et al., 2021; Vishram et al., 2021; Carbonari et al., 2022). Instead, O91 was detected three times less frequently than the emerging O80 serogroup across our collection of human isolates (Crombé and Piérard, 2021). For the food and animal reservoirs, the distribution of serogroups could be evaluated over multiple years since the collection representative for what circulates in both reservoirs was covering a larger time period (i.e., from 2014 onwards) compared to those of the human reservoir (i.e., only from 2018 onwards). An emergence of O113 in food and of O157 and O146 in animals was observed over the years. Interestingly, these results were not observed at European level in the yearly zoonosis reports of EFSA [European Food Safety Authority (EFSA), 2019, 2021, 2022; European Food Safety Authority (EFSA), and European Centre for Disease Prevention and Control (ECDC), 2021], where O157 was mostly determined as the highest prevalent serogroup each year across both reservoirs. This is potentially the effect of the previous focus on testing for the O157 serogroup only, whereas laboratories only started having increased awareness for non-O157 serogroups in more recent years.

For the five serogroups most frequently detected across the human collection in this study, their prevalence in the food and/or animal reservoir was investigated. Both animals and foods were determined to be important reservoirs for these serogroups. The most frequent animal reservoir in which the five serogroups were found was cattle, followed by other ruminants (i.e., sheep and goats). Source attribution studies show the importance of cattle, which is generally recognized as the natural host for STEC bacteria (Venegas-Vargas et al., 2016), as the main direct and indirect source of human disease (Mughini-Gras et al., 2018c). Aside from cattle, other studies have also isolated multiple serogroups associated with human infections from sheep (Heuvelink et al., 1998; Urdahl et al., 2003) and goats (Pritchard et al., 2000). Meats and their products from bovine origin, but also milk products were determined to be the main food reservoirs in which these serogroups circulate according to our collection. Indeed, this food reservoir was previously mentioned to be frequently involved in human STEC infections (Murphy et al., 2007; Mughini-Gras et al., 2018c; Adams et al., 2019). The fact that these products were mainly bovine related is again in line with the general knowledge of cattle being the main STEC reservoir. Attributing reservoirs to STEC strains that often cause human disease can help in directing sampling strategies and increasing awareness of certain reservoirs. Moreover, these studies are important to be performed at the national level to identify the best prevention strategies for each country, in addition to those mentioned in European legislations, since transmission pathways were previously determined to be country-specific due to, e.g., cultural (eating) habits (Kintz et al., 2017).

Although WGS uplifts serotyping to the next level compared to conventional methods since it enables determining the serotype of almost all STEC strains, the importance of the serotype in human disease is decreasing since STEC pathogenicity was determined to be more dependent on the isolates’ virulence profiles. Indeed, other studies have noted that the historical importance of defining and classifying STEC isolates according to their serotype in general for risk assessment and other purposes is decreasing (Boss and Hummerjohann, 2019; Werber and Scheutz, 2019), because of the plasticity of the STEC genome resulting in the same serotypes carrying different genetic characteristics (Koutsoumanis et al., 2020). Because WGS also enables complete virulence genotyping, it is a perfect method to use for STEC pathogenicity studies as well. Indeed, by linking the virulence potential of isolates with the severity of the clinical outcome in corresponding cases (i.e., defined by the incidence of HUS [EFSA Panel on Biological Hazards (BIOHAZ), 2013)], multiple association studies were previously conducted to facilitate pathogenicity prediction of STEC isolates (Koutsoumanis et al., 2020). Also in this study, the prevalence of virulence genes was investigated across human isolates with a severe (i.e., HUS cases) and less severe disease (i.e., no HUS cases) outcome. A collection of 36 virulence genes was determined to be more prevalent in severe cases and significant associations to severe illness were obtained for toxin gene *stx2, espI* and *cif*. When looking at gene subtypes for the *stx* and *eae* genes, *stx2a* and *eae-β* were significantly associated with severe illness. According to our results, these genes and gene subtypes can therefore be regarded as general indicator genes for severe disease. Other studies were able to identify associations for half of these 36 virulence genes (Donohue-Rolfe et al., 2000; Mundy et al., 2004; Bugarel et al., 2010a; Buvens and Piérard, 2012; De Boer et al., 2015; Franz et al., 2015; Matussek et al., 2017; Naseer et al., 2017; Koutsoumanis et al., 2020; Bai et al., 2021) and even extended it further to others whose gene products function as toxins, adhesins, autotransporters, in invasion, and in the secretion system. For the other half of these 36 virulence genes (n: 18; i.e. *iha, ehxA, cba, subA, cma, capU, aggA, aggB, aggC, aggD, aggR, iroN, pic, sepA, aaiC, aap, aar*, and *aatA*) this is, to the best of our knowledge, the first study describing a higher prevalence in severe vs. less severe disease. However, more studies are required in the future to investigate their potential role in and association with severe STEC-related illness, because insufficient data were available in this study to do so. Moreover, since all these 36 genes were also represented in less severe cases across our collection, they could not be defined as definitive markers for STEC pathogenicity. From the *stx2* variants for which sufficient data were available, variants *stx2a* and *stx2c* were determined to be associated with severe and mild disease, respectively. Other studies have indeed also confirmed the higher potency of *stx2a* to cause severe disease (Ethelberg et al., 2004; Buvens and Piérard, 2012; De Rauw et al., 2018b), and a low potency of *stx2c* similar to that of *stx1* to cause HUS (Fuller et al., 2011). De Rauw et al. (2018a) could also determine an association of *stx2d* with severe disease which could not be detected here because of its low prevalence across the human collection for which metadata were available. We could confirm the significant link of *stx1* to milder disease. As also described in other studies (Koutsoumanis et al., 2020), *stx1a* was the most frequent variant in human disease according to our study. In almost all cases, *stx1* presence in HUS cases was accompanied by *stx2* and a dozen of other virulence genes that might have contributed to the strain’s pathogenicity. In the two solely *stx1*-positive strains, the corresponding cases were aged under five at the time of the infection, making them more vulnerable to the development of severe illness. In one of these two cases, the solely *stx1*-positive strain was, moreover, accompanied by another isolate carrying both *stx1* and *stx2* genes that could have also contributed to the HUS development.

The vast majority of STEC isolates causing HUS are known to possess adherence factors, such as the product of the intimin *eae* gene, that enable the bacterial isolate to attach to human intestinal cells (Koutsoumanis et al., 2020). Only one isolate causing severe illness in this study was indeed identified to not carry any attachment gene. Of all *eae*-positive isolates in this study, *eae-γ1* was the main subtype of *eae* circulating across the human isolates for which metadata on their clinical outcome was available (i.e., in 60.1% of *eae* positive isolates). Another study found an association between this variant and severe disease (Hua et al., 2020). However, a similar association was not observed in this study, based on a larger collection of isolates with the *eae-γ1* variant [i.e., n: 140 in this study vs. 68 in the other (Hua et al., 2020)]. On the contrary, variant *eae-γ1* was even determined to be slightly more prevalent, although not significantly, in less severe illnesses compared to in HUS cases. Moreover, despite its lower prevalence across the collection of human isolates for which clinical metadata were available, an association with serious illness could be identified for *eae-β*. This variant was already described previously to be related to virulent serotypes (i.e., O26:H11; Koutsoumanis et al., 2020), but this is the first study in which an association of the intimin subtype with severe disease could be determined. Although still more research should be done on the potential association of the other variants with severe disease, these results already show the importance of *eae* variant subtyping, which is possible with the routine application of WGS. Moreover, the results also partly contribute to the data gap mentioned by the EFSA 2020 report on STEC pathogenicity assessment, concerning the role of *eae* variants in severe disease (Koutsoumanis et al., 2020). Although our study enabled to identify certain virulence genes and/or variants that are more prevalent in HUS cases, our results further support (Koutsoumanis et al., 2020) that it is likely the combination of different virulence genes that determines the potency of a strain to cause severe illness (Koutsoumanis et al., 2020). Aside from STEC-related virulence genes, also virulence genes associated with other *E. coli* pathotypes were detected. A high prevalence of these cross-pathotype STEC strains was determined in our study, which is in line with the emerging trend observed across literature (Scheutz et al., 2011; Nyholm et al., 2015). These strains often exhibit a different phenotype affecting their pathogenicity [e.g., the hybrid O104:H4 strain causing the large Germany outbreak in 2011 (Scheutz et al., 2011)]. With the ability of WGS to enable complete virulence genotyping, it also serves as a perfect tool to investigate the pathogenicity of these strains as well. However, it has to be kept in mind that the pathogenic ability of STEC strains is also influenced by the general health and immune status of the affected host. Because of reasons to protect the patient hospital data and this information not being available as standard in the questionnaires taken from the involved human cases during infection, we could not take into account the health status of the patient in this study. It was moreover not the main goal of our study to investigate STEC pathogenicity. Based on determined associations for STEC pathogenicity, a comprehensive approach for prioritization of STEC isolates with certain characteristics can eventually be defined and put in place in terms of public health protection. Currently, the EFSA advises to report STEC based on *stx* gene presence solely [EFSA Panel on Biological Hazards (BIOHAZ), 2013; Koutsoumanis et al., 2020], whereas the Food and Agriculture Organization (FAO) and the World Health Organization (WHO) insist upon a prioritization of STEC isolates based on virulence gene combinations and associations with clinical severity (Food and Agriculture Organization of the United Nations, and World Health Organization, 2018). With regard to the latter, Lindqvist et al. have developed a new approach for ranking of STEC genotypes, combining the estimated probability of the strain to cause severe illness with the public health burden associated with the illness in terms of Disability-adjusted life years or DALY per case (Lindqvist et al., 2023). At the moment, each country determines its own STEC reporting policy in agreement with the minimal requirements described by the EU food legislation. Once more genomic data are generated from the routine WGS implementation, more extensive risk assessment studies can be performed and a prioritization approach can be set up at a national level by the competent authorities.

WGS moreover also enables complete AMR genotyping, through which the corresponding AMR phenotype can be predicted. Multiple studies have indeed demonstrated a high concordance between AMR susceptibility testing and WGS-based phenotype prediction for STEC (Lindsey et al., 2016; Holmes et al., 2018; Gentle et al., 2019). In our study, the majority of the isolates had no predicted AMR. However, AMR resistance was also observed to differ between reservoirs, with a higher predicted AMR prevalence in the food reservoir. Other studies have mainly observed high AMR prevalence in animal isolates (Meng et al., 1998; Stephan and Schumacher, 2001; Vidovic et al., 2013). However, the potential reasons for the difference in AMR prevalence between the reservoirs remain unknown (Mir and Kudva, 2019). One hypothesis applicable to our study is the observed serogroup-dependence of circulating AMR genes. Indeed, antibiotic resistance was more common in non-O157 strains, which is in line with other literature studies (Schroeder et al., 2002; Sasaki et al., 2012; Mir and Kudva, 2019). Since O157 was the most prevalent serogroup in our human collection, it likely contributed to the increased predicted antibiotic susceptibility that was observed in human isolates. Moreover, the combination of O157 with O146 (which was also observed to have low predicted AMR prevalence) as important serogroups in the animal reservoir is likely the reason for the high AMR susceptibility across animal isolates that was observed in this study. For the isolates in which AMR resistance was predicted, it was mainly against aminoglycosides, sulphonamides, tetracyclines, trimethoprims and β-lactams. These findings are in agreement with previous results from other countries worldwide (Amézquita-López et al., 2016; Mukherjee et al., 2017; Zhang et al., 2018; Jafari et al., 2021). All these antibiotic groups are important for medical purposes in livestock animals and diverse human infections (Buvens et al., 2012; van Duijkeren et al., 2019). Moreover, β-lactams and tetracyclines were previously considered as antibiotics of first choice in Europe for growth promotion in food-producing animals (Manden, 1963). Both aspects likely resulted in selection pressure favoring strains that are resistant to these antibiotic groups. The majority of the isolates with predicted AMR resistance were identified to confer resistance to more than one antibiotic group, among which all ESBL bacteria. Alarmingly, this was also seen in other studies (Ahmed and Shimamoto, 2015; Ranjbar et al., 2018; Elmonir et al., 2021; Joseph and Kalyanikutty, 2022). ESBL as well as colistin resistance are forming global threats to antibiotic treatment possibilities. However, colistin resistance was only very limitedly represented in STEC isolates in this study. Interestingly, the overall prevalence of AMR across the collection in this study was decreasing over the past few years. While studies across the world based on older collections (i.e., before 2014) still described an increasing tendency of AMR prevalence over the years (Mukherjee et al., 2021), a more recent study from the UK analyzing STEC strains from 2014 to 2016 was able to show a more stable course in AMR prevalence for the first time (Gentle et al., 2019). Our study is the first to describe a declining trend of AMR prevalence in STEC in this area. Although more longitudinal research based on large representative collections is still required, these results might indicate that the efforts in stimulating justifiable use of antibiotics in humans and animals are starting to pay off.

Routinely applying WGS in food safety as well as public health surveillance of STEC enables the investigation of the presence and frequency of SNPs or other mutations in certain genes thanks to its single nucleotide resolution. In our study, we used the example of the stress response *rpoS* gene that was previously determined to possess mutations in human isolates stimulating its survival in the human tract (van Hoek et al., 2013; Franz et al., 2015). Moreover, the stress response system plays an important role in risk assessment of STEC bacteria (Koutsoumanis et al., 2020). We found a slightly higher percentage of human isolates containing a *rpoS* variant compared to isolates of bovine origin, which is in line with the conclusion of van Hoek et al. (2013) and Franz et al. (2015). However, the vast majority of the variants detected in our study were determined to be synonymous. Synonymous codon mutations were long thought to be silent. However, a growing body of evidence now shows that they can have effects on codon usage (Parvathy et al., 2022) and protein folding (Liu, 2020). Therefore, to provide further input to the eventual effect of these *rpoS* mutations, more research is required to investigate the influence of these mutations at protein level.

Although valuable information was generated in this study showing the strength of applying WGS in food safety as well as in public health surveillance, the efficiency of WGS in detecting genomic relationships and elaborating STEC epidemiology is dependent on the representativeness and maturity of the surveillance system in place. We reported several clusters containing only human isolates with no genomic relationship toward isolated animal or food strains. Moreover, multiple serotypes (such as, e.g., O80:H2) were only detected in specific reservoirs, without representation in the other reservoirs. Similarly, also detected EAEC-STEC isolates in the collection were only represented across the human reservoir, without any coverage of the cross-pathotype across other reservoirs. Therefore, our results likely indicate that the current dataset applied in this study potentially has gaps in identifying all circulating threats in foods and animals that cause disease in humans. A first cause of this gap could be the applied isolation methods, differences in their use between the entities involved in national STEC surveillance in public health and food context (e.g., the NRL and NRC STEC; see Supplementary material) and their sensitivity to enable STEC isolation. Indeed, the absence of detecting certain serotypes or characteristics in specific reservoirs might be related, aside from prevalence differences, to the serotype-specific isolation approaches often used as methods for STEC surveillance (Nouws et al., 2020a). If these methods differ, the potential and diversity of STEC serotypes to be isolated might be affected and the comparability between both bodies might be impacted. Although very difficult because of the differences in sample matrices and level of sample contaminations, optimizing applied isolation methods and harmonizing them between involved entities would likely increase the comparability and representativeness of isolated strains from the different matrices. On the other hand, not all clusters of human cases should necessarily link or are challenging to link back to a causal strain from animal or food origin. Indeed, clusters might consist of human cases that got ill after traveling abroad, might have been caused by person-to-person contact, or might be difficult to trace back because of international and free foodstuff export in combination with diet diversity (Wahl et al., 2011; Byrne et al., 2015; Scavia et al., 2018). The free foodstuff export and its accompanying increased frequency of cross-border outbreaks demonstrate the importance of WGS implementation for surveillance by all countries globally and that these data are all uploaded to a centralized database on a real-time basis, to enable quick outbreak management. The limited availability of metadata concerning human cases and the origin of food matrices in this study forms a potential second cause describing the difficulties in identifying the causal threats of human disease. A third cause is linked to the applied STEC collection being incomplete. Indeed, the collection in this study is based on a selection of isolates obtained in the scope of surveillance, i.e., not necessarily on all isolates circulating in Belgium. For the human collection, not all human isolates causing disease in Belgium and often only those that caused severe disease are centralized, despite the recommendation to send all. For the food and animal collection, not all isolates from the official control samples, which is moreover only a sample of the population circulating across Belgium, were included in the collection (see Supplementary material). The fact that isolates picked up from animal carcasses were defined as originating from the animal reservoir in this collection (see Supplementary material) might also be biased. Some characteristics might be over-or underrepresented in the collection, because of the inclusion of multiple isolates of the same cluster within the collection, because of the applied isolation methods that are often serotype-specific (as is already mentioned as a first cause describing the gap in the dataset), and because of the O157 serogroup being a selection criterion during the setup of the food and animal collection (see Supplementary material). Lastly, another possible reason describing the gap in the current dataset would be the level of representativeness of the sampling strategies in place with regard to the national epidemiological context. Also the EFSA stated that mature sampling strategies are rarely obtained within countries due to biased sample availability and lack of sampling of putative infection sources (Thépault et al., 2018; Mughini-Gras et al., 2018a), but that they are highly required to ensure representativeness and statistical power of the dataset [EFSA BIOHAZ Panel (EFSA Panel on Biological Hazards) et al., 2019]. Although other studies have already shown the importance of mature monitoring systems and extensive questionnaires (Ingelbeen et al., 2018; Thépault et al., 2018; Mughini-Gras et al., 2018a), the EFSA emphasized the need for studies defining these statistically powerful and representative sampling strategies. With the goal to contribute to this requirement, we attempted to provide different suggestions that can aid in further optimizing the STEC food safety surveillance system at a national level. Firstly, aside from the official control samples, centralizing all self-checking samples at a laboratory with WGS capacity would largely increase the number of isolates available for sequencing. In Belgium, a similar recommendation already exists for public health STEC surveillance to gather all samples or isolates of human origin. Secondly, expanding STEC surveillance toward other reservoirs can also increase the number and diversity of STEC isolates obtained. The reservoirs that are preferable subject to be included in sampling strategies can be different from country to country, based on cultural habits. As an example, currently no surveillance is performed on the environmental reservoir in Belgium. However, tap water, including well water, was determined to be the third main source of strong evidence outbreaks of STEC across Europe in 2020 (Koutsoumanis et al., 2020). The environment and applied tools and utensils within slaughterhouses, food preparation industries or butchers have already been implicated in causing the produced food to be contaminated and entering the food chain (Wilson et al., 2018; Cap et al., 2019; Babolhavaeji et al., 2021). The environment of petting zoos and farms, but also direct contact with the housed animals, have been determined to be important causes of STEC infections in Europe (Warshawsky et al., 2002; Conrad et al., 2017; Schlager et al., 2018). With these examples, including the environmental reservoir in routine surveillance is suggested in an attempt to further optimize sampling strategies. Moreover, self-checking samplings within the primary production reservoir, i.e., animals that are bred for human consumption or whose products are consumed, is not mandatory in Belgium (FAVV-AFSCA, 2019). Re-introducing these samplings into the animal reservoir would also aid in maturing the surveillance system in place. To give a more specific example; serogroup O80 which was determined to be one of the major serogroups causing human disease according to the applied collection, was also observed to be emerging across other European countries (Wijnsma et al., 2017; Ingelbeen et al., 2018; Nüesch-Inderbinen et al., 2018). However, aside from its determined presence in diarrheic calves that are not used for human consumption, its reservoirs and different transmission routes to humans are still unclear (Ingelbeen et al., 2018). Increasing the samplings to different reservoirs, can help in identifying the reservoirs of O80 isolates as well. Thirdly, adapting the European guidelines to mandate more frequent samplings will also generate a more representative targeting of circulating STEC strains. Currently, monitoring data for STEC in foods and animals in Europe originate from the reporting obligations under Directive 2003/99/EC (European Union, 2003). However, this Directive does not explicitly address sampling strategy or sampling frequencies. Although it remains difficult to define strategies at European level due to cultural differences between countries, it would be feasible to recommend an increase in sampling frequency and to improve harmonization of sampling investigations between countries. However, it is currently difficult to make specific recommendations on the number of strains that need to be sampled to achieve relevant surveillance. Other studies have shown that even a large number of shared genomes only allow linkage to a limited number of strains, and even then this only concerned *Salmonella* and *Listeria* (Nielsen et al., 2017; Sanaa et al., 2019). As STEC are driven by strong evolutionary forces, strain diversity is potentially more difficult to measure. This difficulty needs to be taken into account as it may temper the expectation of the promise of genomics. However, after a few years of WGS-based monitoring of the different reservoirs, it would be interesting to quantitatively measure the diversity captured (using rarefaction curves) in order to further optimize the sampling plan and define a specific number of required samplings per reservoir to obtain a representative collection of circulating STEC strains.

Although WGS implementation in routine food safety surveillance requires substantial investments, public health profits will likely result from the improved sampling strategies and implementation of WGS. Multiple economic analyses have shown already a public health benefit of WGS implementation in routine food safety practices that exceeds those of the investments required for its application (Jain et al., 2019; Brown et al., 2021) because WGS allows rapidly picking up signals and preventing outbreaks or at least limit their burdens. Considering this, it is up to the policy makers to explore how to financially enable WGS implementation.

In conclusion, by demonstrating the benefits of implementing WGS in routine food safety surveillance with specific examples, we delivered evidence-based results to encourage the competent authorities to support this process. Moreover, with the analysis of 754 WGS data sets, our study further contributed to the development of a representative WGS-based collection of circulating strains in Belgium that can even be added to the joint EFSA-ECDC database [European Centre for Disease Control (ECDC), European Food Safety Authority (EFSA) et al., 2019]. As shown in this study, centralizing all WGS-based results with corresponding metadata from different reservoirs and sectors indeed helps national and international players in the field to rapidly detect genomic links and respond quickly in case of food safety threats. Moreover, to be able to fully exploit the benefits offered by WGS, suggestions to further optimize current sampling strategies were given. WGS is currently being implemented in multiple countries worldwide, which is of global food safety importance. It is therefore critical to follow this trend and to upload all genomic data and accompanying metadata on a real-time basis to a centralized database, also for food safety surveillance, to optimize prevention and management of national and international outbreaks.

## Data availability statement

The datasets presented in this study can be found in online NCBI SRA [i.e. BioProject accession number PRJNA574887 (Nouws et al., 2020b), PRJNA633966 (Bogaerts et al., 2021), PRJNA645975 (Nouws et al., 2020a), and PRJNA936486] and ENA (i.e. PRJEB60270) repositories. The names of the repository/repositories and accession number(s) can be found in the Supplementary Table S1.

## Ethics statement

The isolates of human origin that were used in this study were all collected in the frame of routine public health surveillance from fecal samples. No additional testing was performed on any of these fecal samples. The accompanying metadata were collected retrospectively from patient charts and anonymously stored in a database in the frame of Decision No 2119/98/EC of the European Parliament and of the Council, concerning the epidemiological surveillance and control of communicable diseases in the Community, as completed by Decision No 1082/2013/EU. As no additional sampling or information was asked from patients, no formal approval from an ethical committee or informed consents were needed. Conclusively, ethical review and approval was not required for the study on human participants in accordance with the local legislation and institutional requirements. Written informed consent for participation was not required for this study in accordance with the national legislation and the institutional requirements.

## Author contributions

SN and SDK conceived and designed the study, with contributions from NR, SD, and BV, interpreted the results, and wrote the manuscript. SDK supervised the project. SD and BV were responsible for the initial isolation and sharing of food and animal isolates together with their corresponding metadata. SN performed the wet lab experiments in preparation of the sequencing of all animal and food isolates. FC and DP were responsible for the initial isolation, the subsequent wet lab experiments and WGS of all human STEC isolates, and the sharing of their WGS and metadata. BB and KV provided the bioinformatics advice and applied tools in Galaxy. SN performed the data analysis. BV, SD, FC, DP, BB, KV, KM, and NR provided specialized feedback on the obtained results. All authors contributed to the article and approved the submitted version.

## Funding

This research was funded by Sciensano (contract RP. Be READY) and the Belgian Federal Public Service of Health, Food Chain Safety and Environment (contract RF 17/6316 StEQIDEMIC.be).

## Conflict of interest

The authors declare that the research was conducted in the absence of any commercial or financial relationships that could be construed as a potential conflict of interest.

## Publisher’s note

All claims expressed in this article are solely those of the authors and do not necessarily represent those of their affiliated organizations, or those of the publisher, the editors and the reviewers. Any product that may be evaluated in this article, or claim that may be made by its manufacturer, is not guaranteed or endorsed by the publisher.

## Footnotes

^1^The views and opinions of the authors expressed herein do not necessarily state or reflect those of the European Center for Disease prevention and Control (ECDC). The accuracy of the authors’ statistical analysis and the findings they report are not the responsibility of ECDC. ECDC is not responsible for conclusions or opinions drawn from the data provided. ECDC is not responsible for the correctness of the data and for data management, data merging and data collation after provision of the data. ECDC shall not be held liable for improper or incorrect use of the data.
