# Supplementary material for: Transforming Shiga toxin-producing Escherichia coli surveillance through whole genome sequencing in food safety practices
Source: Front Microbiol. 2023 Jul 13;14:1204630. doi: 10.3389/fmicb.2023.1204630 (PMC10381951; doi:10.3389/fmicb.2023.1204630)
Supplement: Supplementary file 2 [file Data_Sheet_1.pdf]

Supplementary Material

Transforming Shiga toxin-producing *Escherichia coli* surveillance through Whole Genome Sequencing in food safety practices

Stéphanie Nouws[1,2], Bavo Verhaegen[3], Sarah Denayer[3], Florence Crombé[4], Denis Piérard[4], Bert Bogaerts[1], Kevin Vanneste[1], Kathleen Marchal[2,5], Nancy H. C. Roosens[1], and Sigrid C. J. De Keersmaecker[1*]

[1]Transversal activities in Applied Genomics, Sciensano, Brussels, Belgium

[2]IDlab, Department of Information Technology, Ghent University - IMEC, Ghent, Belgium

[3]National Reference Laboratory for Shiga toxin-producing *Escherichia coli* (NRL STEC) and for Foodborne Outbreaks (NRL FBO), Foodborne Pathogens, Sciensano, Brussels, Belgium

[4]National Reference Centre for Shiga toxin-producing *Escherichia coli* (NRC STEC), Universitair Ziekenhuis Brussel, Vrije Universiteit Brussel, Brussels, Belgium

[5]Department of Plant Biotechnology and Bioinformatics, Ghent University, Ghent, Belgium

*** Correspondance:**
Sigrid De Keersmaecker
Sigrid.DeKeersmaecker@Sciensano.be

Keywords: Whole Genome Sequencing$_1$, Shiga toxin-producing *Escherichia coli*$_2$, Surveillance$_3$, Food safety$_4$, Implementation$_5$.

1 Supplementary Materials and Methods

1.1 Inclusion criteria for isolates within the STEC collection

To set up the collection applied in this study, isolates were mainly selected from those obtained in the scope of the national STEC surveillance system covering the different STEC reservoirs, to obtain a STEC collection representative for the circulating background in Belgium. To do so, all strains from official control samples across the food chain (received by the Belgian National Reference Laboratory of STEC (NRL STEC)) between 2012 and 2020 were included. However, from the official control samples taken from animal carcasses within the same time period, only the STEC isolates of bovine origin, all STEC O157 isolates of caprine and ovine origin, and 10.0% of the STEC non-O157 isolates of caprine and ovine origin were included in this study. Strains isolated from animal (bovine, ovine and caprine) carcasses were considered as originating from the animal reservoir, since bacterial isolates in feces and on hides of cattle were previously described to be a source for carcass contamination (Cummings et al., 2010; Croxen et al., 2013; Gutema et al., 2021). To further complete the collection with isolates sampled in the scope of the national STEC surveillance system, all strains at the NRL STEC isolated from self-checking samples taken by different farmers across Belgium between 2012 and 2020 were also included. Additional isolates from human cases available at the NRL STEC because they were shared previously by the Belgian entity analyzing human samples and isolates (i.e. the

National Reference Centre of STEC (NRC STEC)) in the scope of outbreak investigations were also included in the collection. Of all these selected isolates, only those for which a pure culture glycerol-stock (in which the isolates are preserved) was available and for which the isolates could be confirmed as being STEC (through PCR detection of the housekeeping *uidA* gene and the STEC-defining *stx* gene, as described in the paragraph on sample preparation and sequencing in the main manuscript) were eventually included.

This collection was further supplemented with WGS data of human isolates obtained from public health surveillance between 2018 and 2020 (including a limited number of isolates from the beginning of 2021), processed and sequenced by the NRC STEC. Despite the recommendation to send all clinical isolates to the NRC-STEC, not all isolates circulating across humans in Belgium are centralized there. At the NRC STEC, isolation (according to an isolation method different from the one used by the NRL STEC4) and sequencing was performed on a routine basis since 2019 (also retrospectively), and assemblies are publicly available at EnteroBase ((Zhou et al., 2020); https://enterobase.warwick.ac.uk/). For this study, the raw WGS reads were shared with us.

Public WGS data of other studies, containing a collection of human, food and animal isolates representative of what was circulating in Belgium between 1998 and 2014 (n: 139; (Nouws et al., 2020b; Bogaerts et al., 2021)) and all isolates involved in the two major Belgian STEC outbreaks of the last decade (n: 43; (Nouws et al., 2020a)), were added to this selection. Also for these studies, food and animal isolates were originating from the NRL STEC, whereas human isolates originated from the NRC STEC.

Of all these selected isolates, the in-house produced, publicly available, and shared WGS data were only included in the final collection, when:

(I) Data analysis confirmed the corresponding isolates as being STEC (through WGS-based detection of *stx* and *E. coli* confirmation with Kraken2 analysis, as described in the paragraph of WGS data analysis in the main manuscript);

(II) Contaminant reads of bacterial species other than *E. coli* defined as low-level contaminations (≤ 5.9 %) and detected by Kraken2 analysis (≤ 5.9 % of the detected reads) could be removed (as described in the paragraph of WGS data analysis in the main manuscript);

(III) No WGS data quality errors were detected (as described in the paragraph of WGS data analysis in the main manuscript);

(IV) No within-species contaminations were suspected;

An overview of the selection process of all isolates or their corresponding WGS data included within this study is shown in Supplementary Figure 1.

For the remainder of this paragraph, isolates originating from animal carcasses or animal feces were defined as coming from the animal reservoir, strains isolated from food samples across the complete food chain were defined as coming from the food reservoir, and isolates from human cases were defined under the human reservoir.

1.2 Performed prevalence studies

To deliver insight on the different STEC strains circulating in Belgium, multiple prevalence studies were performed. Any of these studies were based on a part of the collection being representative of the circulating STEC strains in Belgium, i.e. containing the majority of isolates obtained from the surveillance system. In other words, the prevalence of characteristics in the food, animal, or human reservoir was calculated on a sub-collection of these reservoir-specific isolates covering 2014 to 2020 (for food and animal isolates) or 2018 to 2020 (for human isolates, not including the limited number of strains isolated in the beginning of 2021). Before (or after) these time periods, only a part of the isolates picked up by surveillance systems were available to be included in the study collection. Indeed, no representative collection of the official control samples taken before 2014 were physically available at the NRL STEC for the food/animal isolates and no representative collection of human isolates was sequenced at the NRC STEC before 2018. Therefore, no prevalence studies can be performed before 2014 (for food/animal isolates) or 2018 (for human isolates). When comparing the prevalence of certain characteristics between reservoirs, this was done between the same time periods, i.e. from 2018 to 2020 (not including the limited number of strains isolated in 2021). When describing the prevalence of specific characteristics across the reservoirs (e.g. which *stx* variants were the most prevalent across the collection), the collection from 2018 to 2020 (not including the limited number of strains isolated in 2021) was employed because it contains the best representation of circulating strains within each reservoir. Supplementary table S6 summarizes the time periods and number of isolates in the reservoirs for which the prevalence of certain characteristics was compared. Only when the interest was in calculating the prevalence of a characteristic (e.g. AMR) in isolates with another specific characteristic (e.g. only in those with serotype O157:H7), the sub-collection used for the prevalence study included all isolates within the entire collection that contained the specific characteristic (e.g. all isolates with serotype O157:H7).

2 References

Bogaerts, B., Nouws, S., Verhaegen, B., Denayer, S., Van Braekel, J., Winand, R., et al. (2021). Validation strategy of a bioinformatics whole genome sequencing workflow for Shiga toxin-producing *Escherichia coli* using a reference collection extensively characterized with conventional methods. *Microb Genom* 7. doi: 10.1099/mgen.0.000531.

Croxen, M. A., Law, R. J., Scholz, R., Keeney, K. M., Wlodarska, M., and Finlay, B. B. (2013). Recent Advances in Understanding Enteric Pathogenic *Escherichia coli*. *Clin Microbiol Rev* 26, 822–880. doi: 10.1128/CMR.00022-13.

Cummings, K. J., Warnick, L. D., Elton, M., Gröhn, Y. T., McDonough, P. L., and Siler, J. D. (2010). The Effect of Clinical Outbreaks of Salmonellosis on the Prevalence of Fecal *Salmonella* Shedding Among Dairy Cattle in New York. *Foodborne Pathog Dis* 7, 815–823. doi: 10.1089/fpd.2009.0481.

Gutema, F. D., Agga, G. E., Abdi, R. D., Jufare, A., Duchateau, L., De Zutter, L., et al. (2021). Assessment of Hygienic Practices in Beef Cattle Slaughterhouses and Retail Shops in Bishoftu, Ethiopia: Implications for Public Health. *Int J Environ Res Public Health* 18, 2729. doi: 10.3390/ijerph18052729.

Nouws, S., Bogaerts, B., Verhaegen, B., Denayer, S., Crombé, Fl., De Rauw, K., et al. (2020a). The Benefits of Whole Genome Sequencing for Foodborne Outbreak Investigation from the

Perspective of a National Reference Laboratory in a Smaller Country. *Foods* 9, 1030. doi: 10.3390/foods9081030.

Nouws, S., Bogaerts, B., Verhaegen, B., Denayer, S., Piérard, D., Marchal, K., et al. (2020b). Impact of DNA extraction on whole genome sequencing analysis for characterization and relatedness of Shiga toxin-producing *Escherichia coli* isolates. *Sci Rep* 10, 14649. doi: 10.1038/s41598-020-71207-3.

Zhou, Z., Alikhan, N.-F., Mohamed, K., Fan, Y., and Achtman, M. (2020). The EnteroBase user's guide, with case studies on *Salmonella* transmissions, *Yersinia pestis* phylogeny, and *Escherichia* core genomic diversity. *Genome Res* 30, 138–152. doi: 10.1101/gr.251678.119.
