# Supplementary material for: Transforming Shiga toxin-producing Escherichia coli surveillance through whole genome sequencing in food safety practices
Source: Front Microbiol. 2023 Jul 13;14:1204630. doi: 10.3389/fmicb.2023.1204630 (PMC10381951; doi:10.3389/fmicb.2023.1204630)
Supplement: Supplementary file 3 [file Image_1.pdf]

Supplementary Material

Transforming Shiga toxin-producing *Escherichia coli* surveillance through Whole Genome Sequencing in food safety practices

Stéphanie Nouws[1,2], Bavo Verhaegen[3], Sarah Denayer[3], Florence Crombé[4], Denis Piérard[4], Bert Bogaerts[1], Kevin Vanneste[1], Kathleen Marchal[2,5], Nancy H. C. Roosens[1], and Sigrid C. J. De Keersmaecker[1*]

[1]Transversal activities in Applied Genomics, Sciensano, Brussels, Belgium

[2]IDlab, Department of Information Technology, Ghent University - IMEC, Ghent, Belgium

[3]National Reference Laboratory for Shiga toxin-producing *Escherichia coli* (NRL STEC) and for Foodborne Outbreaks (NRL FBO), Foodborne Pathogens, Sciensano, Brussels, Belgium

[4]National Reference Centre for Shiga toxin-producing *Escherichia coli* (NRC STEC), Universitair Ziekenhuis Brussel, Vrije Universiteit Brussel, Brussels, Belgium

[5]Department of Plant Biotechnology and Bioinformatics, Ghent University, Ghent, Belgium

*** Correspondance:**
Sigrid De Keersmaecker
Sigrid.DeKeersmaecker@Sciensano.be

Keywords: Whole Genome Sequencing[1], Shiga toxin-producing *Escherichia coli*[2], Surveillance[3], Food safety[4], Implementation[5].

1 Supplementary Figure S1: Overview of selection process and workflow of the isolates and WGS data within the STEC collection

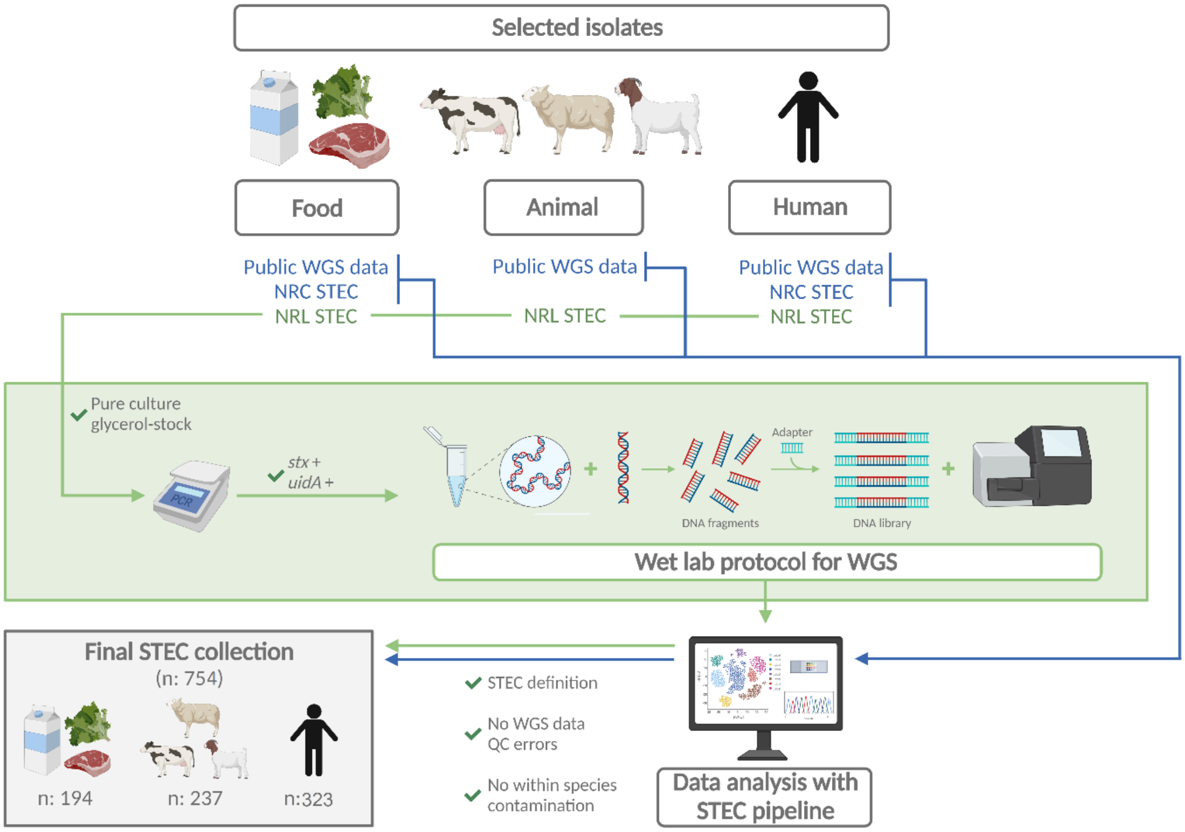

Supplementary Figure S 1. Overview of the selection process and workflow of the isolates and WGS data within the STEC collection. The collection included 754 STEC isolates, from which 194 were from food origin, 237 from animal origin, and 323 from human origin. The animal reservoir contained both strains isolated from animal feces (n: 25) and strains isolated from the animal carcasses (n: 212; since their contamination was considered to have occurred from unsanitary slaughter). These animals included cattle, sheep and goats. When the animal species was unknown, it was indicated as such. When the animal species was uncertain between sheep and goats, it was indicated as 'Ovine/caprine'. This figure was created with BioRender.com (2022), adapted from "NextGenerationSequencingWorkflow" by BioRender.com (2022), and retrieved from https://app.biorender.com/biorender-templates. The collection contained WGS data sets from the NRL STEC, the NRC STEC and from other Belgian studies that are publicly available (Nouws et al., 2020b, 2020a; Bogaerts et al., 2021) to come to a selection covering the food, human and animal reservoirs that is representative for the circulating background in Belgium. The blue arrows show the workflow followed for the publicly available WGS data (i.e. mainly from the NRC STEC and other Belgian studies). As indicated by this workflow, these data were analyzed with the STEC pipeline developed and validated by Bogaerts et al. (Bogaerts et al., 2021), and added to the STEC collection when fulfilling the predefined criteria (i.e. the isolates were defined as STEC through confirmation of *stx* and *E. coli* detection, no errors were detected in the WGS data quality control (QC) check, no contamination was detected and no within-species contamination was detected). The green arrows indicate the workflow followed for all human, food and animal isolates obtained from the NRL STEC, i.e. the isolates were grown from pure culture glycerol-stocks, the presence of STEC and *E. coli* specific

virulence genes (i.e. *stx* and *uidA*) was confirmed with PCR on a heat lysate, and DNA was extracted and prepared (with Nextera XT library preparation) for WGS on the MiSeq. The data were then similarly processed with the STEC pipeline, and added to the STEC collection when fulfilling the same predefined criteria.

2 Supplementary Figure S2: Identified clusters in the O145, O26, O103 and O80 serogroups

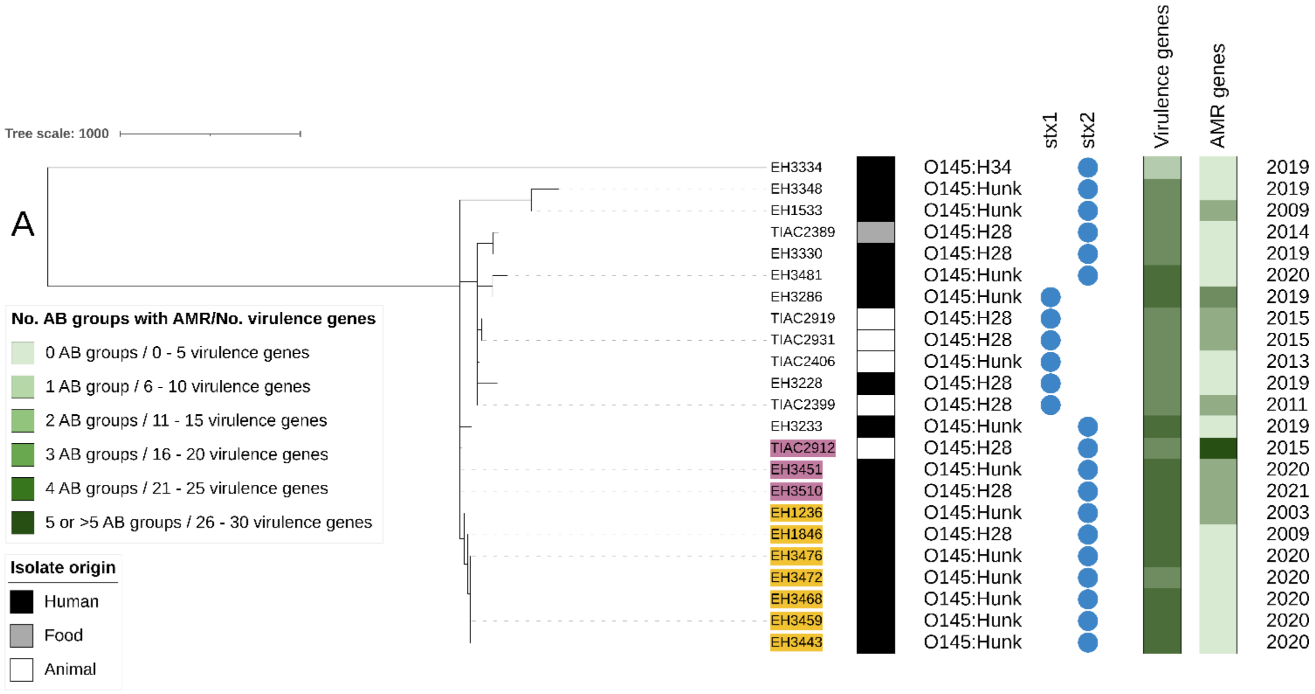

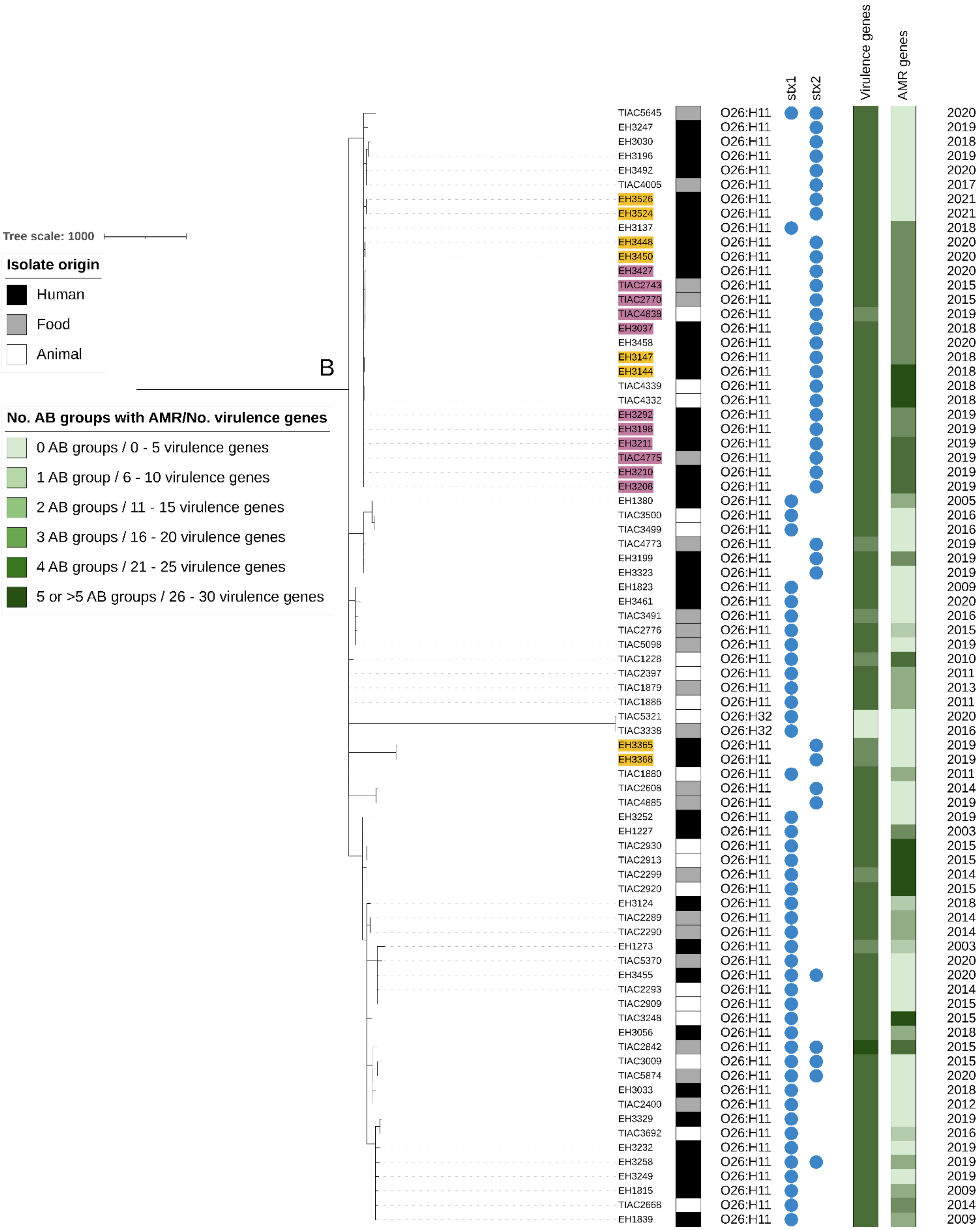
Tree scale: 1000
Isolate origin
Human
Food
Animal
B
No. AB groups with AMR/No. virulence genes
0 AB groups / 0 - 5 virulence genes
1 AB group / 6 - 10 virulence genes
2 AB groups / 11 - 15 virulence genes
3 AB groups / 16 - 20 virulence genes
4 AB groups / 21 - 25 virulence genes
5 or >5 AB groups / 26 - 30 virulence genes
stx1
stx2
Virulence genes
AMR genes
TIAC5645 O26:H11 2020
EH3247 O26:H11 2019
EH3030 O26:H11 2018
EH3196 O26:H11 2019
EH3492 O26:H11 2020
TIAC4005 O26:H11 2017
EH3526 O26:H11 2021
EH3524 O26:H11 2021
EH3137 O26:H11 2018
EH3448 O26:H11 2020
EH3450 O26:H11 2020
EH3427 O26:H11 2020
TIAC2743 O26:H11 2015
TIAC2770 O26:H11 2015
TIAC4838 O26:H11 2019
EH3037 O26:H11 2018
EH3458 O26:H11 2020
EH3147 O26:H11 2018
EH3144 O26:H11 2018
TIAC4339 O26:H11 2018
TIAC4332 O26:H11 2018
EH3292 O26:H11 2019
EH3198 O26:H11 2019
EH3211 O26:H11 2019
TIAC4775 O26:H11 2019
EH3210 O26:H11 2019
EH3208 O26:H11 2019
EH1380 O26:H11 2005
TIAC3500 O26:H11 2016
TIAC3499 O26:H11 2016
TIAC4773 O26:H11 2019
EH3199 O26:H11 2019
EH3323 O26:H11 2019
EH1823 O26:H11 2009
EH3461 O26:H11 2020
TIAC3491 O26:H11 2016
TIAC2776 O26:H11 2015
TIAC5098 O26:H11 2019
TIAC1228 O26:H11 2010
TIAC2397 O26:H11 2011
TIAC1879 O26:H11 2013
TIAC1886 O26:H11 2011
TIAC5321 O26:H32 2020
TIAC3338 O26:H32 2016
EH3365 O26:H11 2019
EH3368 O26:H11 2019
TIAC1880 O26:H11 2011
TIAC2608 O26:H11 2014
TIAC4885 O26:H11 2019
EH3252 O26:H11 2019
EH1227 O26:H11 2003
TIAC2930 O26:H11 2015
TIAC2913 O26:H11 2015
TIAC2299 O26:H11 2014
TIAC2920 O26:H11 2015
EH3124 O26:H11 2018
TIAC2289 O26:H11 2014
TIAC2290 O26:H11 2014
EH1273 O26:H11 2003
TIAC5370 O26:H11 2020
EH3455 O26:H11 2020
TIAC2293 O26:H11 2014
TIAC2909 O26:H11 2015
TIAC3248 O26:H11 2015
EH3056 O26:H11 2018
TIAC2842 O26:H11 2015
TIAC3009 O26:H11 2015
TIAC5874 O26:H11 2020
EH3033 O26:H11 2018
TIAC2400 O26:H11 2012
EH3329 O26:H11 2019
TIAC3692 O26:H11 2016
EH3232 O26:H11 2019
EH3258 O26:H11 2019
EH3249 O26:H11 2019
EH1815 O26:H11 2009
TIAC2668 O26:H11 2014
EH1839 O26:H11 2009

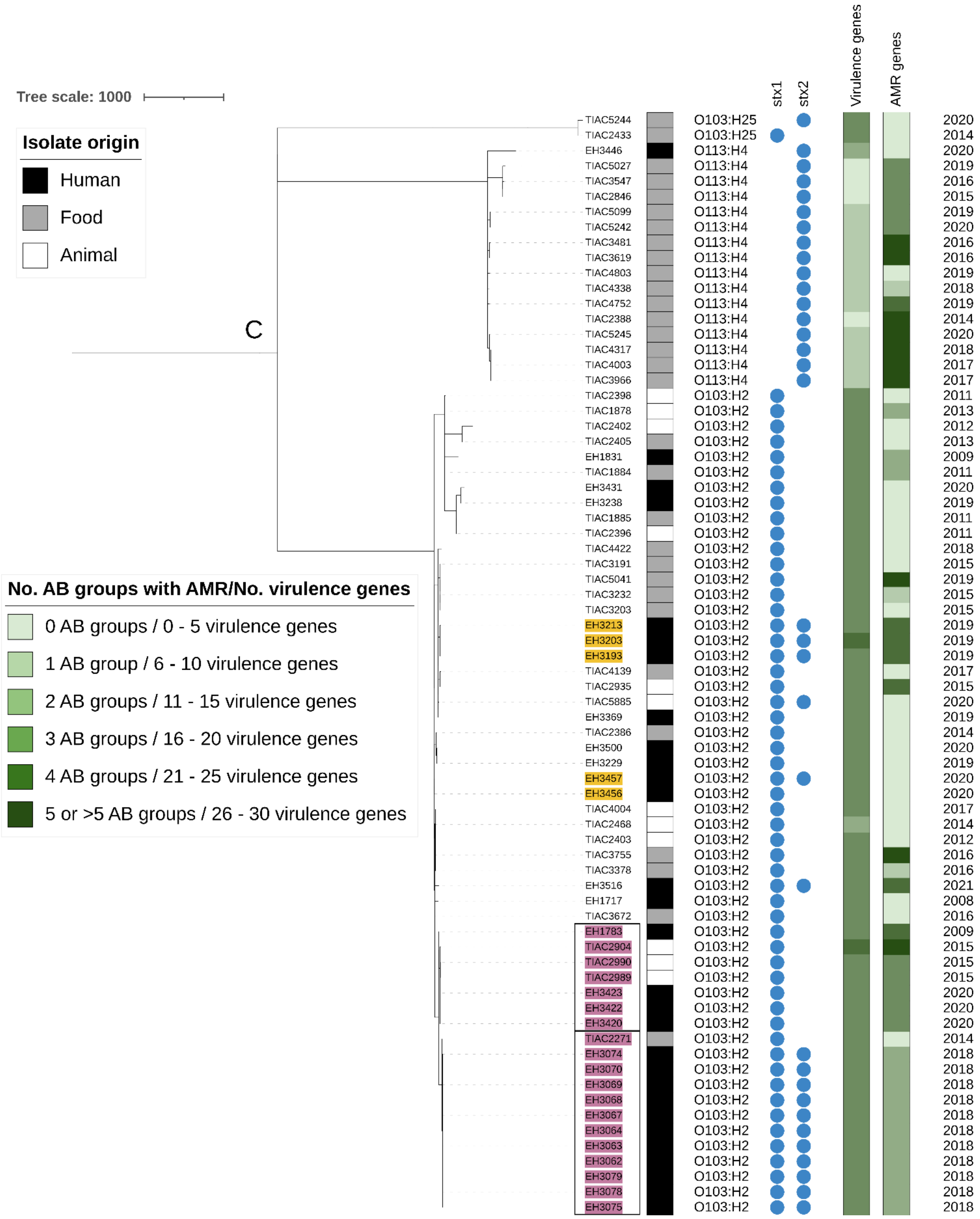

Tree scale: 1000
Isolate origin
Human
Food
Animal
C
No. AB groups with AMR/No. virulence genes
0 AB groups / 0 - 5 virulence genes
1 AB group / 6 - 10 virulence genes
2 AB groups / 11 - 15 virulence genes
3 AB groups / 16 - 20 virulence genes
4 AB groups / 21 - 25 virulence genes
5 or >5 AB groups / 26 - 30 virulence genes
stx1
stx2
Virulence genes
AMR genes
TIAC5244 O103:H25 2020
TIAC2433 O103:H25 2014
EH3446 O113:H4 2020
TIAC5027 O113:H4 2019
TIAC3547 O113:H4 2016
TIAC2846 O113:H4 2015
TIAC5099 O113:H4 2019
TIAC5242 O113:H4 2020
TIAC3481 O113:H4 2016
TIAC3619 O113:H4 2016
TIAC4803 O113:H4 2019
TIAC4338 O113:H4 2018
TIAC4752 O113:H4 2019
TIAC2388 O113:H4 2014
TIAC5245 O113:H4 2020
TIAC4317 O113:H4 2018
TIAC4003 O113:H4 2017
TIAC3966 O113:H4 2017
TIAC2398 O103:H2 2011
TIAC1878 O103:H2 2013
TIAC2402 O103:H2 2012
TIAC2405 O103:H2 2013
EH1831 O103:H2 2009
TIAC1884 O103:H2 2011
EH3431 O103:H2 2020
EH3238 O103:H2 2019
TIAC1885 O103:H2 2011
TIAC2396 O103:H2 2011
TIAC4422 O103:H2 2018
TIAC3191 O103:H2 2015
TIAC5041 O103:H2 2019
TIAC3232 O103:H2 2015
TIAC3203 O103:H2 2015
EH3213 O103:H2 2019
EH3203 O103:H2 2019
EH3193 O103:H2 2019
TIAC4139 O103:H2 2017
TIAC2935 O103:H2 2015
TIAC5885 O103:H2 2020
EH3369 O103:H2 2019
TIAC2386 O103:H2 2014
EH3500 O103:H2 2020
EH3229 O103:H2 2019
EH3457 O103:H2 2020
EH3456 O103:H2 2020
TIAC4004 O103:H2 2017
TIAC2468 O103:H2 2014
TIAC2403 O103:H2 2012
TIAC3755 O103:H2 2016
TIAC3378 O103:H2 2016
EH3516 O103:H2 2021
EH1717 O103:H2 2008
TIAC3672 O103:H2 2016
EH1783 O103:H2 2009
TIAC2904 O103:H2 2015
TIAC2990 O103:H2 2015
TIAC2989 O103:H2 2015
EH3423 O103:H2 2020
EH3422 O103:H2 2020
EH3420 O103:H2 2020
TIAC2271 O103:H2 2014
EH3074 O103:H2 2018
EH3070 O103:H2 2018
EH3069 O103:H2 2018
EH3068 O103:H2 2018
EH3067 O103:H2 2018
EH3064 O103:H2 2018
EH3063 O103:H2 2018
EH3062 O103:H2 2018
EH3079 O103:H2 2018
EH3078 O103:H2 2018
EH3075 O103:H2 2018

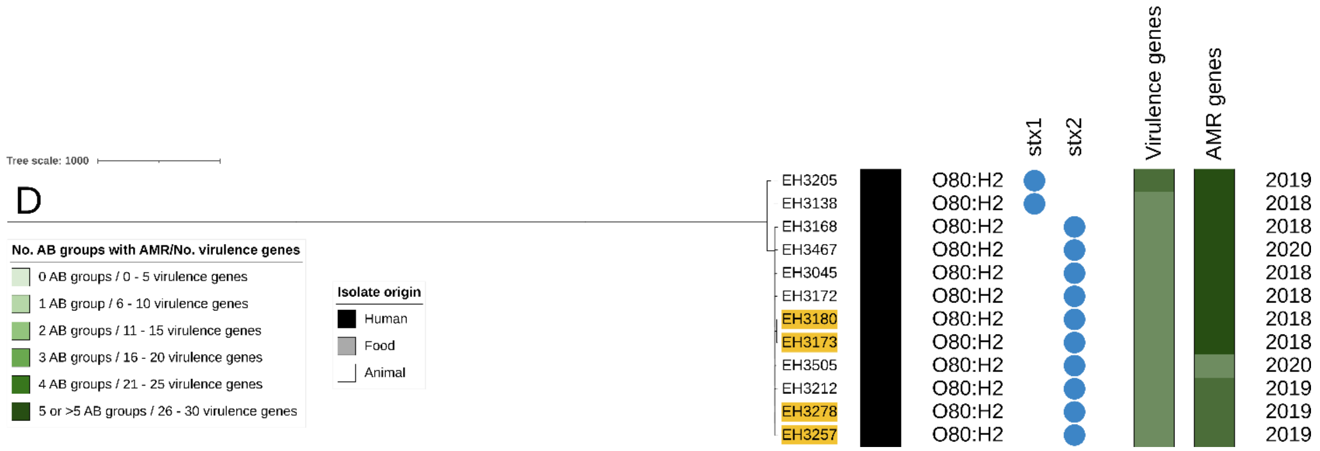

Supplementary Figure S 2 Identified clusters in the O145, O26, O103 and 080 serogroup lineages. A minimum spanning tree was made based on the cgMLST allele matrices of all O145 (S2.A), O26 (S2.B), O103 (S2.C) and O80 (S2.D) isolates using the MSTreeV2 method with GrapeTree. The rectangular trees were visualized with iTOL (Letunic and Bork, 2019). The origin of the isolates (as indicated in the corresponding legend), the serotype, the presence of *stx1* and *stx2* genes, the number of virulence genes, the number of antibiotic (AB) groups for which AMR was predicted (as indicated in the corresponding legend), and the year of strain isolation are annotated on the tree. The scale bars represent the number of core genome allele differences. Isolate names marked in purple indicate clusters of human isolates for which a link with the food or animal reservoir was detected in this study. Isolates marked in yellow indicate clusters of human isolates for which no strain in the animal and/or food reservoir that is closely related to the cluster could be detected in this study. The number of cgMLST allele differences between the isolates of the same cluster was ≤22. The higher the number of cgMLST allele differences detected, the higher the time difference (the largest was 17 years difference) between the isolation of both isolates.

3 Supplementary Figure S3: Prevalence of serogroups in the reservoirs

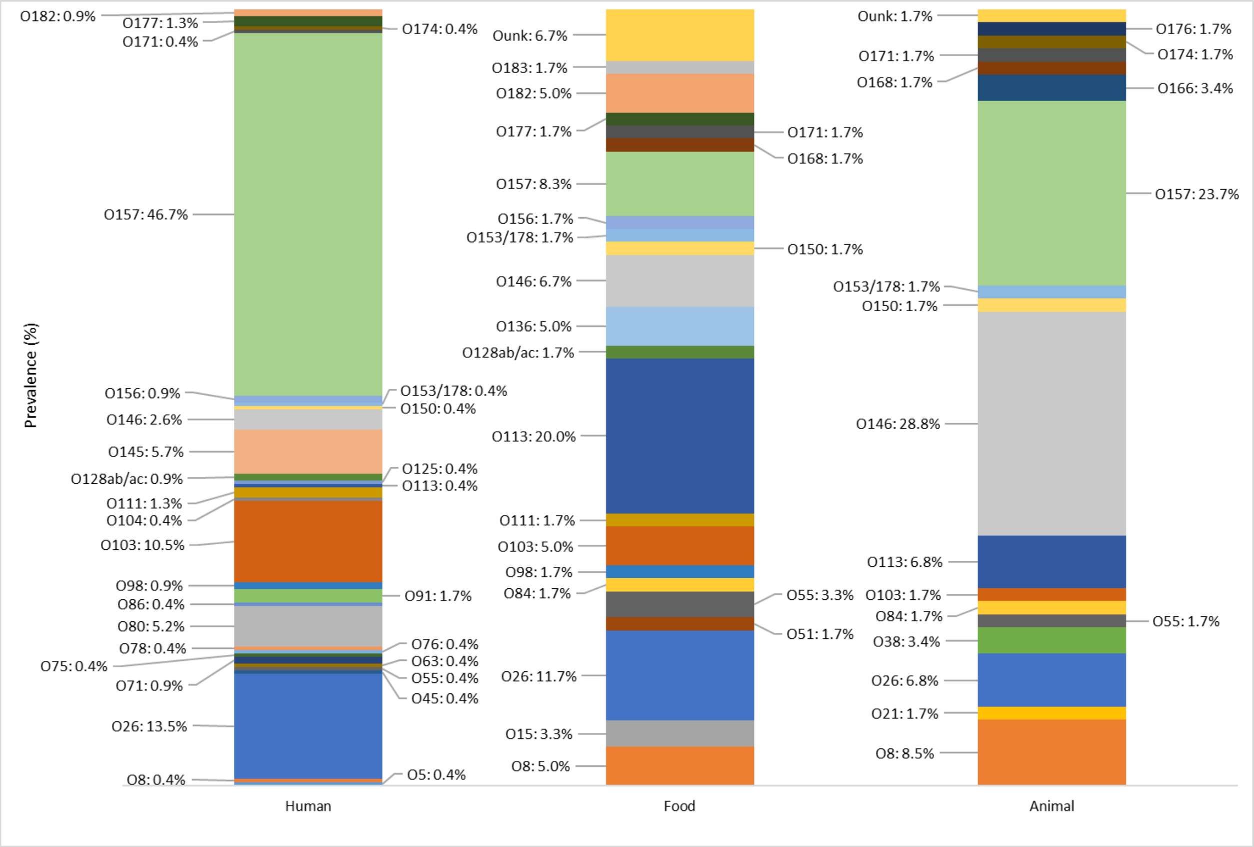

Supplementary Figure S 3. Prevalence of STEC serogroups across the collection of human, food and animal isolates.

4 **Supplementary Figure S4: Prevalence of STEC serogroups in food and animal isolates over the years**

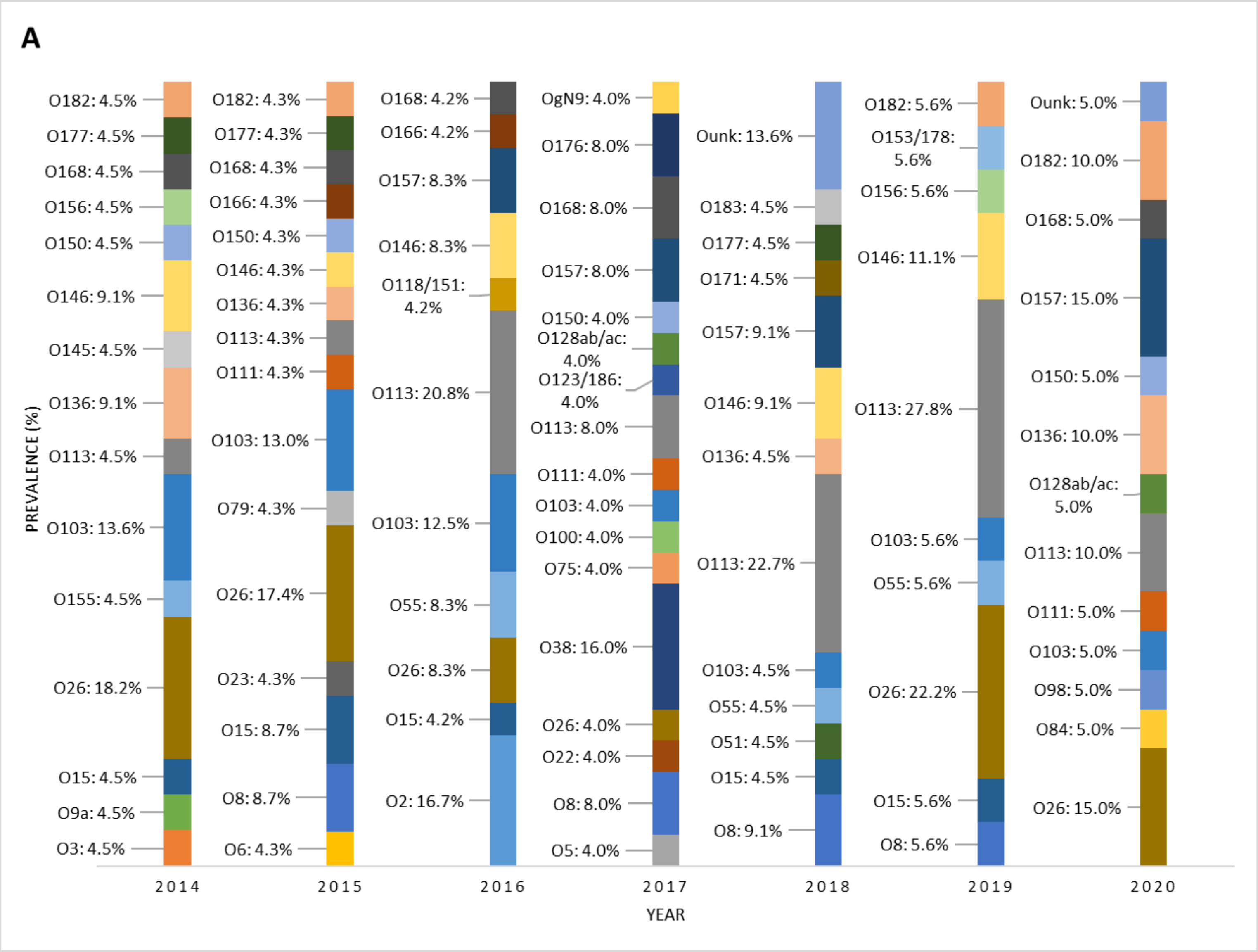

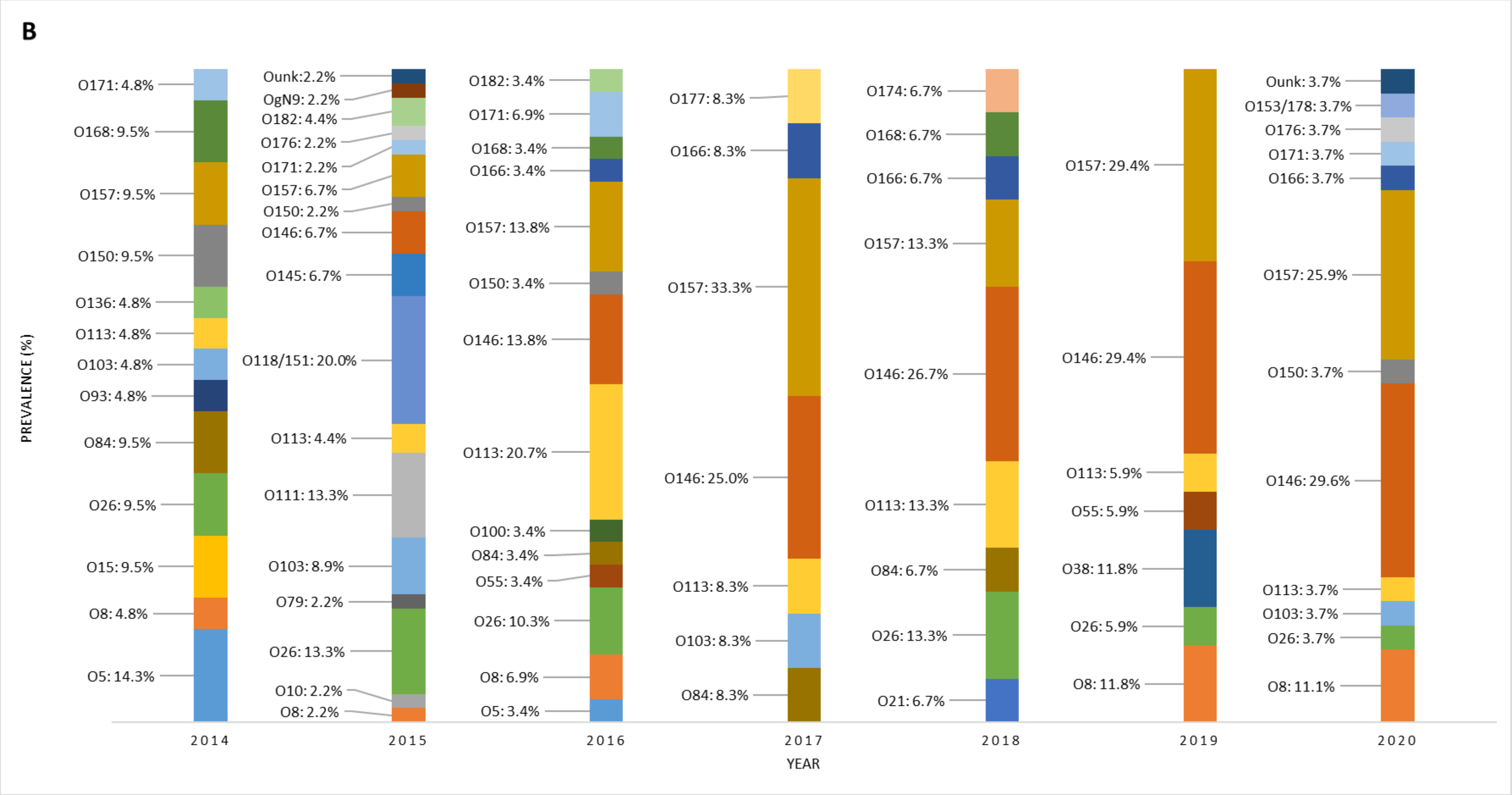

Supplementary Figure S 4. The prevalence of STEC serogroups over the years in the food (S4.a) and animal (S4.b) reservoir.

5 Supplementary Figure S5: Distribution of *stx* and *eae* subtypes in STEC isolates

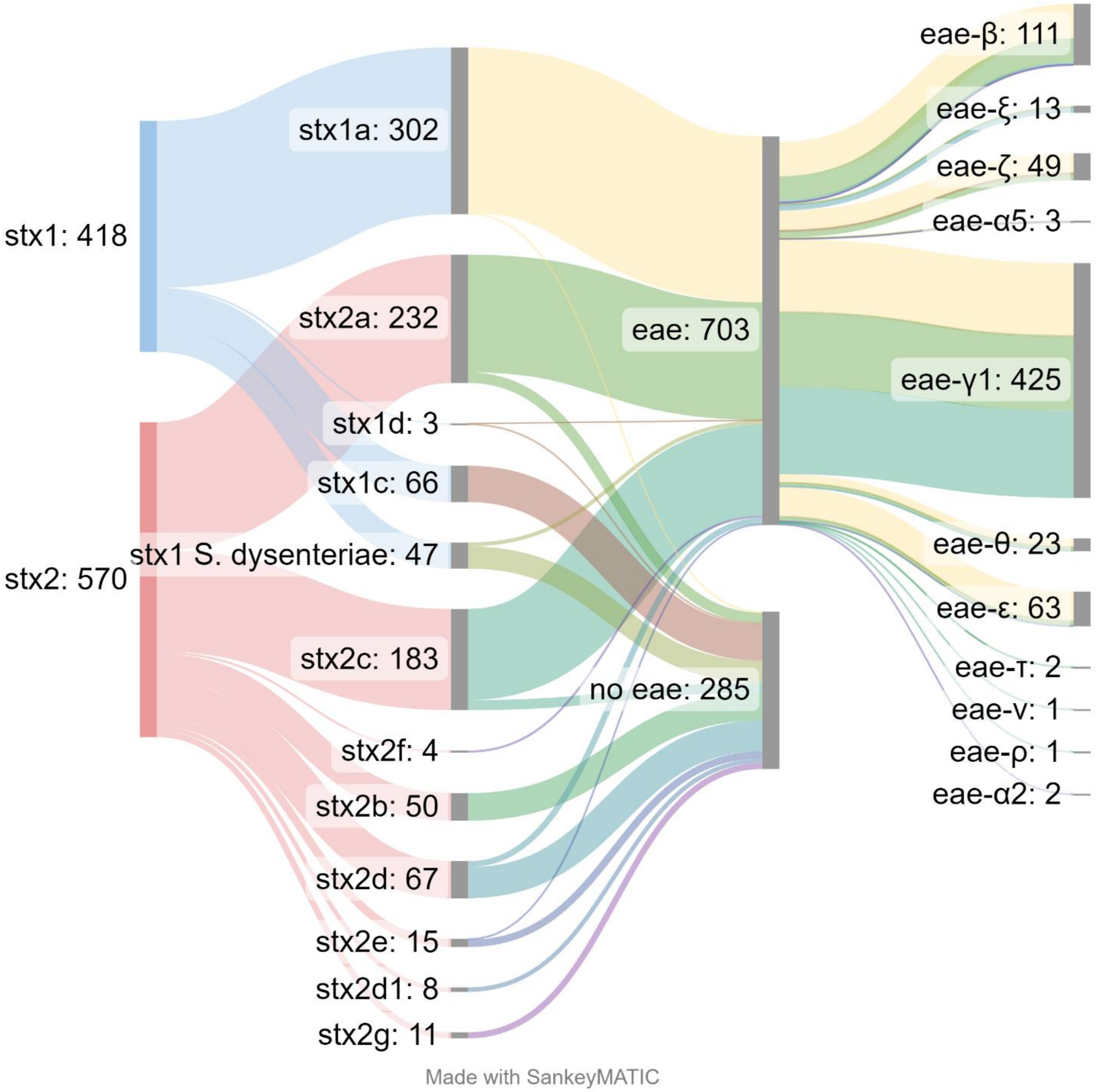

Supplementary Figure S 5. Absolute number of isolates with *stx1* and/or *stx2* and their association with *eae* (subtypes). The Sankey diagram was made with SankeyMATIC (https://sankeymatic.com/build/). The colored flows are *stx* subtype-specific and visualize the number of isolates with that specific *stx* subtype.

6 Supplementary Figure S6: Prevalence of hybrid STEC in the collection

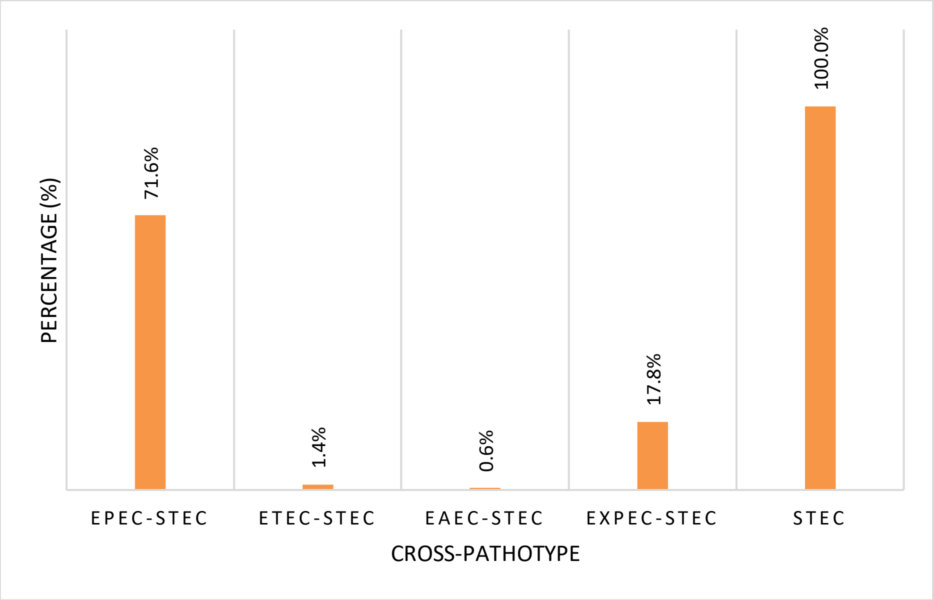

Supplementary Figure S 6. Prevalence of hybrid STEC in the collection.

7 Supplementary Figure S7: Serotypes in severe disease

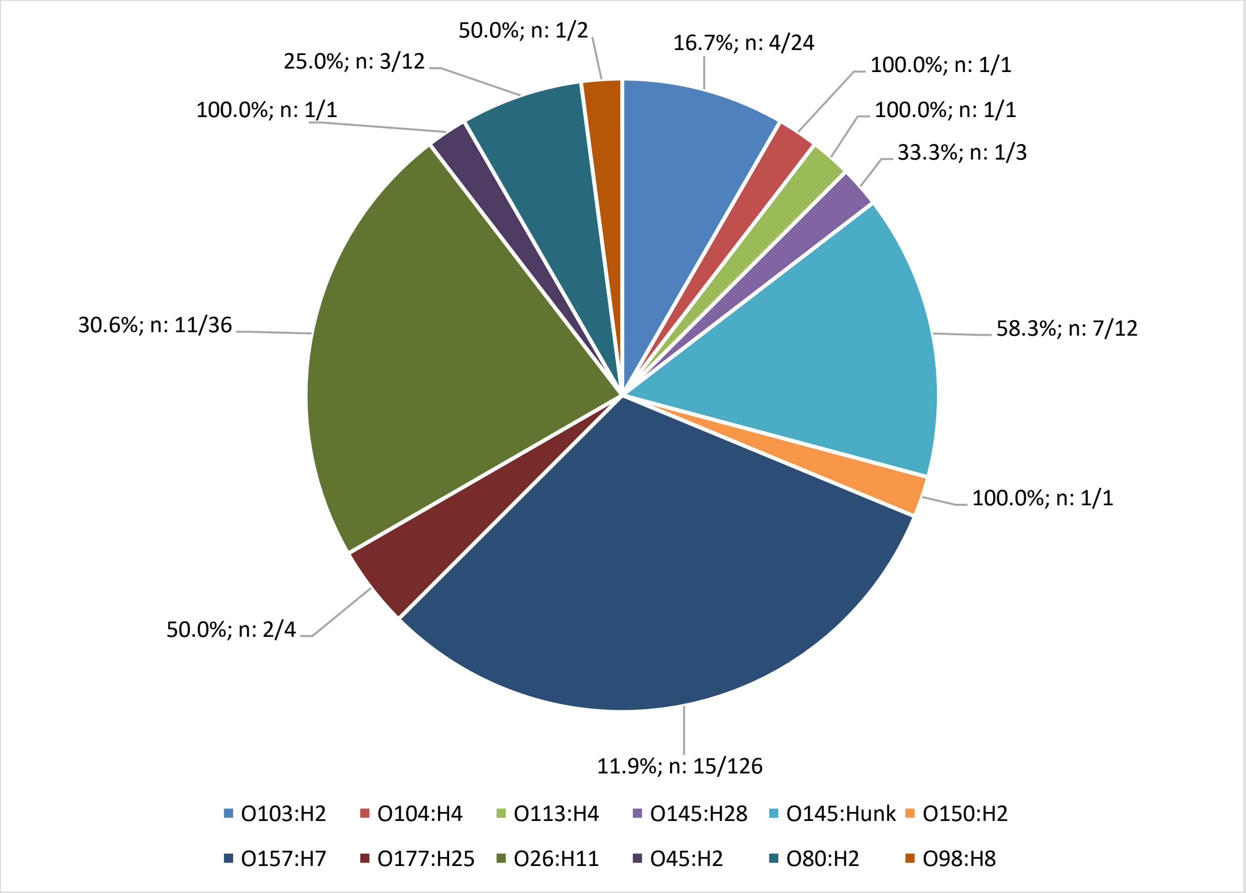

Supplementary Figure S 7. Pie chart visualizing the absolute number of all cases with severe disease per serotype in Belgium across the complete human collection (n: 48, i.e. covering 2003 to 2020). The

percentages next to the chart indicate the percentage of isolates of this serotype that caused severe disease, i.e. percentages above 50.0% indicate that the specific serotype was, based on our collection, more prevalent in severe disease compared to less severe disease and vice-versa. The absolute numbers next to the chart indicate the number of isolates with that specific serotype detected in severe disease over the number of isolates with that specific serotype detected in the sub-collection of human isolates for which metadata were available.

8 Supplementary Figure S8: Prevalence of predicted AMR over the years

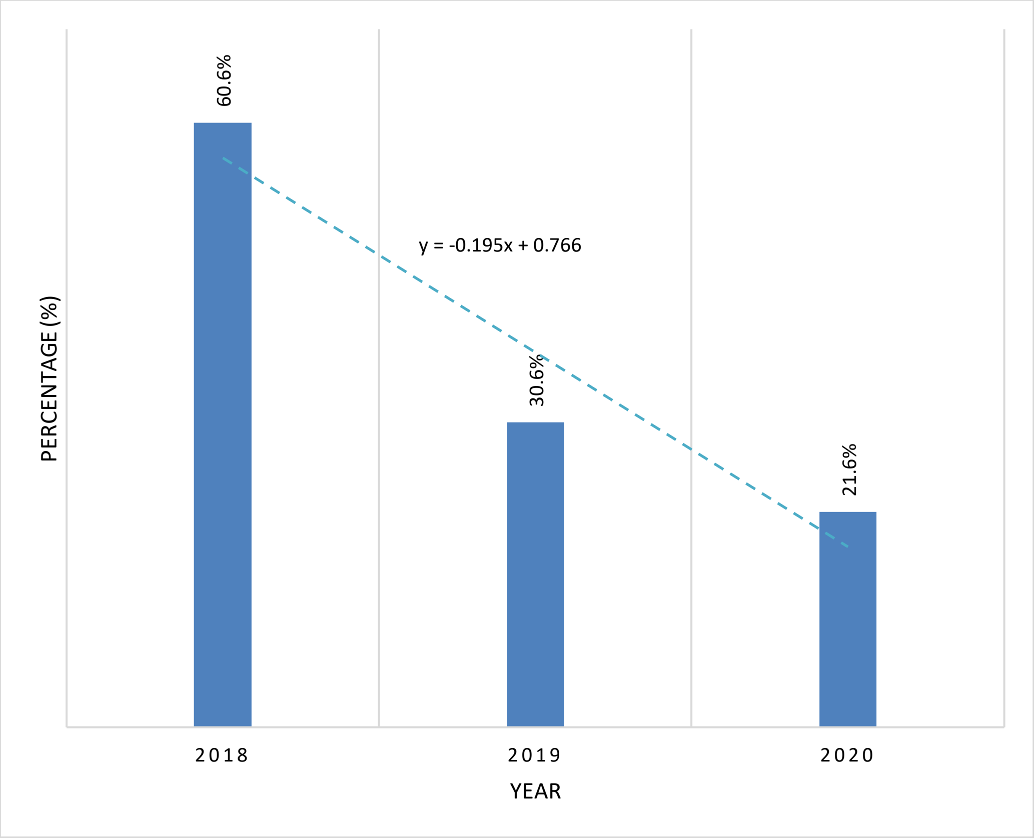

Supplementary Figure S 8. Prevalence of predicted AMR over the years. A trendline with accompanying equation depicts the evolution of predicted AMR over the years. A decreasing trend of AMR is observed.

9 Supplementary Figure S9: Prevalence of predicted AMR over the years per reservoir

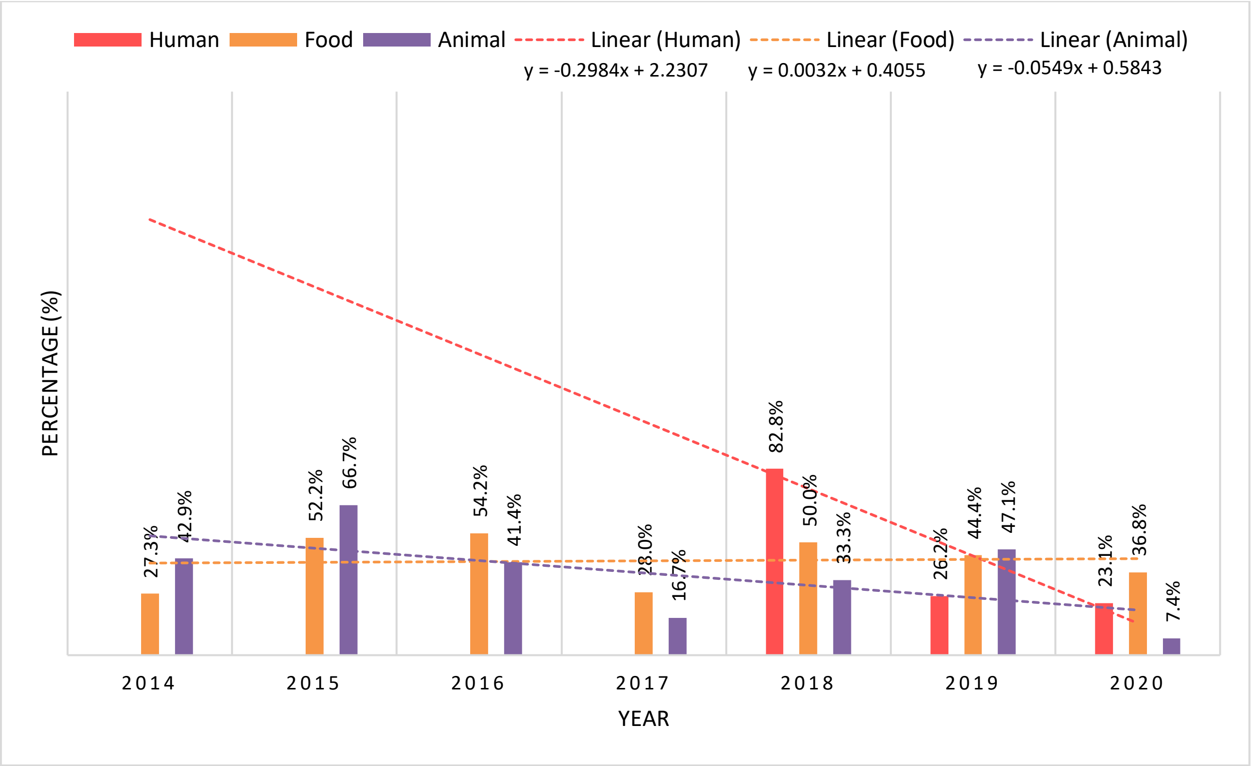

Supplementary Figure S 9. Prevalence of predicted AMR over the years per reservoir. A trendline with accompanying equation depicts the evolution of predicted AMR over the years per reservoir. In the food and human reservoirs a clear decrease in AMR prevalence is observed, whereas a stagnation is observed in the animal reservoir.

10 References

Bogaerts, B., Nouws, S., Verhaegen, B., Denayer, S., Van Braekel, J., Winand, R., et al. (2021). Validation strategy of a bioinformatics whole genome sequencing workflow for Shiga toxin-producing *Escherichia coli* using a reference collection extensively characterized with conventional methods. *Microb Genom* 7. doi: 10.1099/mgen.0.000531.

Letunic, I., and Bork, P. (2019). Interactive Tree Of Life (iTOL) v4: recent updates and new developments. *Nucleic Acids Res* 47, W256–W259. doi: 10.1093/nar/gkz239.

Nouws, S., Bogaerts, B., Verhaegen, B., Denayer, S., Crombé, Fl., De Rauw, K., et al. (2020a). The Benefits of Whole Genome Sequencing for Foodborne Outbreak Investigation from the Perspective of a National Reference Laboratory in a Smaller Country. *Foods* 9, 1030. doi: 10.3390/foods9081030.

Nouws, S., Bogaerts, B., Verhaegen, B., Denayer, S., Piérard, D., Marchal, K., et al. (2020b). Impact of DNA extraction on whole genome sequencing analysis for characterization and relatedness of Shiga toxin-producing *Escherichia coli isolates*. *Sci Rep* 10, 14649. doi: 10.1038/s41598-020-71207-3.
